# Supplementary material for: Feedback regulation of crystal growth by buffering monomer concentration
Source: Nat Commun. 2020 Nov 27;11:6057. doi: 10.1038/s41467-020-19882-8 (PMC7695852; doi:10.1038/s41467-020-19882-8)
Supplement: Supplementary file 1 — Supplementary Information [file 41467_2020_19882_MOESM1_ESM.pdf]

# SUPPLEMENTARY INFORMATION

# Feedback regulation of crystal growth by buffering monomer concentration

Table of Contents


**Supplementary Note 1:** DNA monomer buffering species sequences ....... 2

**Supplementary Note 2:** DNA origami seed design and sequences ....... 3

**Supplementary Note 3:** DNA nanostructured cap design and sequences ....... 9

**Supplementary Note 4:** Modeling unregulated seeded nanotube growth with monomer depletion ....... 12

**Supplementary Note 5:** Modeling unregulated seeded nanotube growth without monomer depletion ....... 14

**Supplementary Note 6:** Determining concentration of viable seeds ....... 18

**Supplementary Note 7:** Determination of the ranges of monomer concentrations corresponding to regimes II (Seeded Nucleation & Growth), III (Growth only) and IV (No Growth) ....... 20

**Supplementary Note 8:** Calculating the equilibrium concentration of active monomers for a set of initial concentrations of buffering species ....... 23

**Supplementary Note 9:** Stochastic kinetic simulations of buffer-regulated nanotube growth ....... 24

**Supplementary Note 10:** Simulations of nanotube growth at a higher setpoint corresponding to $C_i$ = 1.25 µM ....... 32

**Supplementary Note 11:** The reaction rates of monomer buffering may be much slower than the designed rates 34

**Supplementary Note 12:** Growth simulations with higher monomer buffering species concentrations ....... 37

**Supplementary Note 13:** Monomer buffering species affect nanotube growth ....... 38

**Supplementary Note 14:** Image analysis ....... 42

**Supplementary References** ....... 49

## Supplementary Note 1: DNA monomer buffering species sequences

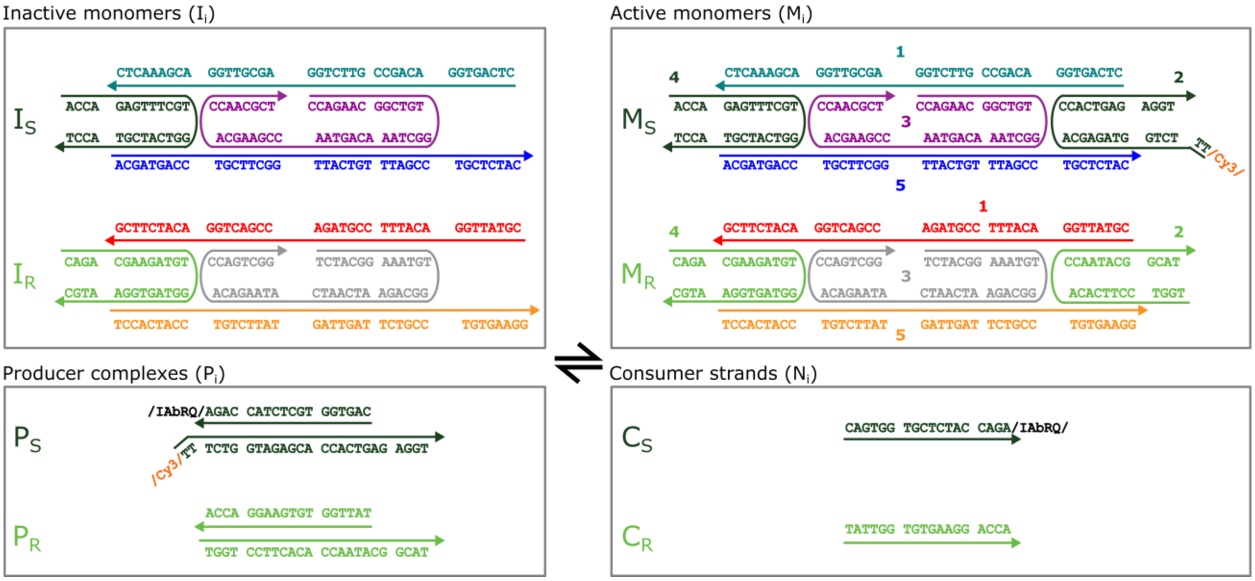


**Supplementary Figure 1:** Design and sequences of the DNA monomer buffering species.

**Supplementary Table 1:** Sequences of monomer and monomer buffering species. The inactive monomers are composed of strands 1, 3, 4, and 5 of their respective active monomers. The Producer complexes are composed of the corresponding Consumer strand and strand 2 of the corresponding active monomer. All strands were ordered HPLC purified from Integrated DNA Technologies, Inc (IDT) and the modifications marked with / / are labeled as defined by IDT.

| Strand name | Sequence |
|---|---|
| $M_R$ – 1 | 5’ CGTATTGGACATTTCCGTAGACCGACTGGACATCTTCG 3’ |
| $M_R$ – 2 | 5’ TGGTCCTTCACACCAATACGGCAT 3’ |
| $M_R$ – 3 | 5’ TCTACGGAAATGTGGCAGAATCAATCATAAGACACCAGTCGG 3’ |
| $M_R$ – 4 | 5’ CAGACGAAGATGTGGTAGTGGAATGC 3’ |
| $M_R$ – 5 | 5’ TCCACTACCTGTCTTATGATTGATTCTGCCTGTGAAGG 3’ |
| $M_S$ – 1 | 5’ CTCAGTGGACAGCCGTTCTGGAGCGTTGGACGAAACTC 3’ |
| $M_S$ – 2 | /5Cy3/TTTCTGGTAGAGCACCACTGAGAGGT 3’ |
| $M_S$ – 3 | 5’ CCAGAACGGCTGTGGCTAAACAGTAACCGAAGCACCAACGCT 3’ |
| $M_S$ – 4 | 5’ ACCAGAGTTTCGTGGTCATCGTACCT 3’ |
| $M_S$ – 5 | 5’ ACGATGACCTGCTTCGGTTACTGTTTAGCCTGCTCTAC 3’ |
| $C_R$ | 5’ TATTGGTGTGAAGGACCA 3’ |
| $C_S$ | 5’ CAGTGGTGCTCTACCAGA/3IAbRQSp/ |

**Supplementary Note 2: DNA origami seed design and sequences**

The design of the DNA origami seed was adopted from previous studies[1,2]. A seed is composed of a scaffold strand (M13mp18 DNA (7,240 bases) purchased from New England Biolabs), 72 staple strands, and 24 adapter strands (strands on the adapters possess the monomer sticky end sequences). Staples that link the top and bottom of the rectangle in the diagram below are a darker shade for clarity. The staple strand sequences are the same as those used in a previous work[1] and include hairpin domains (not depicted in the diagram below) that direct seeds to cyclize with hairpins facing outward, thus assuring the specific direction of curvature that matches that of the nanotubes[1]. The staple sequences are in Supplementary Table 2. The adapter sequences are in Supplementary Table 4.

The fluorescent labeling scheme for the DNA origami seed is the same as a scheme used previously[2]. Briefly, the region of the M13 DNA scaffold not used to fold the cylindrical seed was used as binding sites for one hundred unique DNA strands (termed labeling strands). Each labeling strand has a sequence segment complementary to a portion of the unfolded M13 at its 5' end and also presents a specific 15-base sequence at its 3' end. This 15-base sequence at the 3' end of each labeling strand serves as a binding site for a DNA strand that was modified with a fluorescent tag at its 5' end (either atto488 or atto647 in this study). Thus, each origami seed structure has up to 100 fluorescently labeled strands attached to it. The sequences for the labeling strands are in Supplementary Table 3.

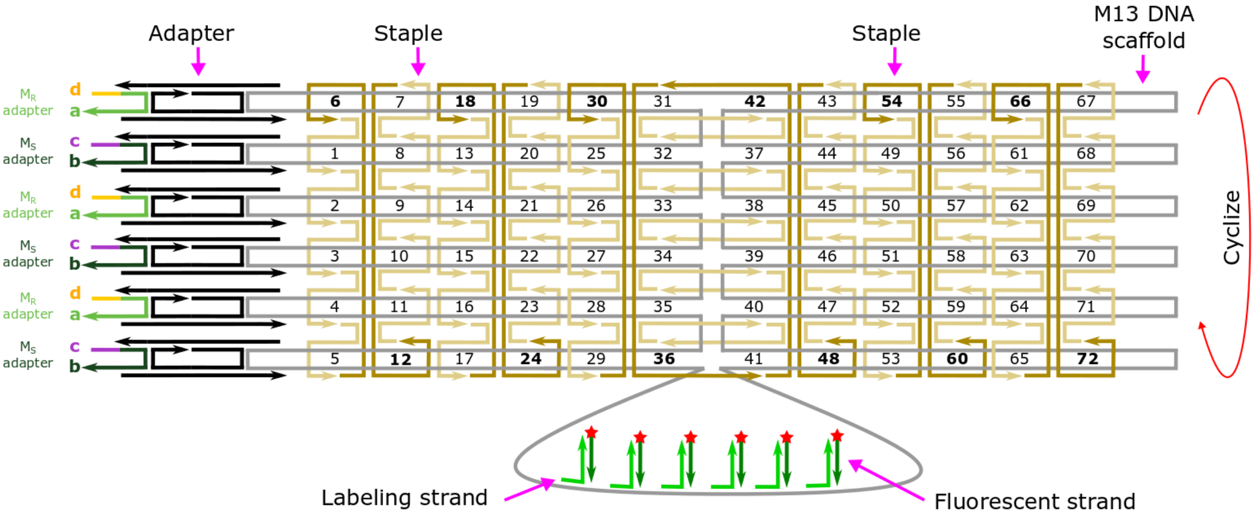


**Supplementary Figure 2:** DNA origami seed design.

**Supplementary Table 2:** Sequences of DNA origami staple strands. All strands ordered unpurified from IDT. Numbers beside strand names correspond to the numbered staples in the diagram in Supplementary Note 2. As previously described, hairpins (highlighted in red) were incorporated into the staples to induce a directional preference for cyclization[1].

| Strand Name | Sequence |
|---|---|
| (1) T_5R2F_HP | 5′ TGAGTTTCAAAGGAACGTCCACCGTTTTCGGTGGACTTAACTAAAGATCTCCAA 3′ |
| (2) T_5R4F_HP | 5′ AAAAAAGGCTTTTGCGGTGGTCCGTTTTCGGACCACTTGGATCGTCGGGTAGCA 3′ |
| (3) T_5R6F_HP | 5′ ACGGCTACAAGTACAACTCGGCACTTTTGTGCCGAGTTCGGAGATTCGCGACCT 3′ |
| (4) T_5R8F_HP | 5′ GCTCCATGACGTAACACGGATCGCTTTTGCGATCCGTTAAGCTGCTACACCAGA 3′ |
| (5) T_5R10F_HP | 5′ ACGAGTAGATCAGTTGCACCGCTGTTTTCAGCGGTGTTAGATTTAGCGCCAAAA 3′ |
| (6) T_5R12F_CYC_HP | 5′ GGAATTACCACCACCCGTGAGGCGTTTTCGCCTCACTTTCATTTTCCGTAACAC 3′ |
| (7) T_5R2E_HP | 5′ GAGAATAGGTCACCAGCGGAACCGTTTTCGGTTCCGTTTACAAACTCCGCCACC 3′ |
| (8) T_5R4E_HP | 5′ AAAGGCCGCTCCAAAACCGTGGCGTTTTCGCCACGGTTGGAGCCTTAGCGGAGT 3′ |
| (9) T_5R6E_HP | 5′ GCGAAACAAGAGGCTTGTGCTGCGTTTTCGCAGCACTTTGAGGACTAGGGAGTT 3′ |
| (10) T_5R8E_HP | 5′ CCAAATCATTACTTAGACGCTGGCTTTTGCCAGCGTTTCCGGAACGTACCAAGC 3′ |
| (11) T_5R10E_HP | 5′ AAAGATTCTAAATTGGCGACGGACTTTTGTCCGTCGTTGCTTGAGATTCATTAC 3′ |
| (12) T_5R12E_CYC_HP | 5′ CTCAGAGCGAGGCATAGGCTCCGCTTTTGCGGAGCCTTGTAAGAGCACAGGTAG 3′ |
| (13) T_3R2F_HP | 5′ TGTAGCATAACTTTCAGGCATCCGTTTTCGGATGCCTTACAGTTTCTAATTGTA 3′ |
| (14) T_3R4F_HP | 5′ TCGGTTTAGGTCGCTGGCTGACGCTTTTGCGTCAGCTTAGGCTTGCAAAGACTT 3′ |
| (15) T_3R6F_HP | 5′ TTTCATGATGACCCCCACCAGCCGTTTTCGGCTGGTTTAGCGATTAAGGCGCAG 3′ |
| (16) T_3R8F_HP | 5′ ACGGTCAATGACAAGACGGAGGCGTTTTCGCCTCCGTTACCGGATATGGTTTAA 3′ |
| (17) T_3R10F_HP | 5′ TTTCAACTACGGAACACTCGCTGCTTTTGCAGCGAGTTACATTATTAACACTAT 3′ |
| (18) T_3R12F_CYC_HP | 5′ CATAACCCACCGCCACCTGGCTCGTTTTCGAGCCAGTTCCTCAGAAACAACGCC 3′ |
| (19) T_3R2E_HP | 5′ TGCTAAACTCCACAGAGCCAGTGCTTTTGCACTGGCTTCAGCCCTCTACCGCCA 3′ |
| (20) T_3R4E_HP | 5′ ATATATTCTCAGCTTGCCGTCCGCTTTTGCGGACGGTTCTTTCGAGTGGGATTT 3′ |
| (21) T_3R6E_HP | 5′ CTCATCTTGGAAGTTTCGGATGGCTTTTGCCATCCGTTCCATTAAACATAACCG 3′ |
| (22) T_3R8E_HP | 5′ AGTAATCTTCATAAGGTCTGGTCGTTTTCGACCAGATTGAACCGAACTAAAACA 3′ |
| (23) T_3R10E_HP | 5′ ACGAACTATTAATCATGGCACCTGTTTTCAGGTGCCTTTGTGAATTTCATCAAG 3′ |
| (24) T_3R12E_CYC_HP | 5′ CCCTCAGATCGTTTACCGCTTGCGTTTTCGCAAGCGTTCAGACGACTTAATAAA 3′ |
| (25) T_1R2F_HP | 5′ CGTAACGAAAATGAATCCTGCCTGTTTTCAGGCAGGTTTTTCTGTAGTGAATTT 3′ |
| (26) T_1R4F_HP | 5′ CTTAAACAACAACCATCGGTGCCGTTTTCGGCACCGTTCGCCCACGCGGGTAAA 3′ |
| (27) T_1R6F_HP | 5′ ATACGTAAGAGGCAAACTCGGTCGTTTTCGACCGAGTTAGAATACACTGACCAA 3′ |
| (28) T_1R8F_HP | 5′ CTTTGAAAATAGGCTGCCGAGGACTTTTGTCCTCGGTTGCTGACCTACCTTATG 3′ |
| (29) T_1R10F_HP | 5′ CGATTTTAGGAAGAAACGGCAGGCTTTTGCCTGCCGTTAATCTACGGATAAAAA 3′ |
| (30) T_1R12F_CYC_HP | 5′ CCAAAATATACTCAGGTGCGGTCGTTTTCGACCGCATTAGGTTTAGATAGTTAG 3′ |
| (31) T_1R2E_HP | 5′ ACGTTAGTTCTAAAGTCGCTTGGCTTTTGCCAAGCGTTTTTGTCGTGATACAGG 3′ |
| (32) T_1R4E_HP | 5′ CAATGACAGCTTGATATGGCGAGCTTTTGCTCGCCATTCCGATAGTCTCCCTCA 3′ |
| (33) T_1R6E_HP | 5′ AAACGAAATGCCACTACCACCTCGTTTTCGAGGTGGTTCGAAGGCAGCCAGCAA 3′ |
| (34) T_1R8E_HP | 5′ CCAGGCGCGAGGACAGCTCTGGACTTTTGTCCAGAGTTATGAACGGGTAGAAAA 3′ |
| (35) T_1R10E_HP | 5′ GGACGTTGAGAACTGGCGAGGCACTTTTGTGCCTCGTTCTCATTATGCGCTAAT 3′ |
| (36) T_1R12E_CYC_HP | 5′ TATCACCGGCGAGAGGCTGCGTCGTTTTCGACGCAGTTCTTTTGCAATCCTGAA 3′ |
| (37) T1R2F_HP | 5′ AGTGTACTATACATGGCTCCTGCGTTTTCGCAGGAGTTCTTTTGATCTTTCCAG 3′ |
| (38) T1R4F_HP | 5′ GAGCCGCCCCACCACCGTCAGGCGTTTTCGCCTGACTTGGAACCGCTGCGCCGA 3′ |
| (39) T1R6F_HP | 5′ AATCACCACCATTTGGCGTCCTGCTTTTGCAGGACGTTGAATTAGACCAACCTA 3′ |
| (40) T1R8F_HP | 5′ TACATACACAGTATGTCGGACCTGTTTTCAGGTCCGTTTAGCAAACTGTACAGA 3′ |
| (41) T1R10F_HP | 5′ ATCAGAGAGTCAGAGGCGAGGTCGTTTTCGACCTCGTTGTAATTGAACCAGTCA 3′ |
| (42) T1R12F_CYC_HP | 5′ TCTTACCATATAAGTACCGAGGCGTTTTCGCCTCGGTTTAGCCCGGAATAGGTG 3′ |
| (43) T1R2E_HP | 5′ TAAGCGTCGGTAATAACAGGAGCGTTTTCGCTCCTGTTGTTTTAACCCGTCGAG 3′ |
| (44) T1R4E_HP | 5′ AACCAGAGACCCTCAGGCAGTCGCTTTTGCGACTGCTTAACCGCCACGTTCCAG 3′ |
| (45) T1R6E_HP | 5′ GACTTGAGGTAGCACCGTCTGGCGTTTTCGCCAGACTTATTACCATATCACCGG 3′ |
| (46) T1R8E_HP | 5′ TTATTACGTAAAGGTGTGGCTGCGTTTTCGCAGCCATTGCAACATACCGTCACC 3′ |
| (47) T1R10E_HP | 5′ TGAACAAAGATAACCCAGTGCCTGTTTTCAGGCACTTTACAAGAATAAGACTCC 3′ |
| (48) T1R12E_CYC_HP | 5′ AGGGTTGAACGCTAACGCCAGGACTTTTGTCCTGGCTTGAGCGTCTGAACACCC 3′ |
| (49) T3R2F_HP | 5′ TGCCTTGACAGTCTCTGTCGGTGCTTTTGCACCGACTTGAATTTACCCCTCAGA 3′ |
| (50) T3R4F_HP | 5′ GCCACCACTCTTTTCACGGTCGGCTTTTGCCGACCGTTTAATCAAATAGCAAGG 3′ |
| (51) T3R6F_HP | 5′ CCGGAAACTAAAGGTGGACCTGGCTTTTGCCAGGTCTTAATTATCATAAAAGAA 3′ |
| (52) T3R8F_HP | 5′ ACGCAAAGAAGAACTGTCGGCTCGTTTTCGAGCCGATTGCATGATTTGAGTTAA 3′ |
| (53) T3R10F_HP | 5′ GCCCAATAGACGGGAGCACAGGCGTTTTCGCCTGTGTTAATTAACTTTCCAGAG 3′ |
| (54) T3R12F_CYC_HP | 5′ CCTAATTTACCAGGCGTCGGAGCGTTTTCGCTCCGATTGATAAGTGGGGGTCAG 3′ |
| (55) T3R2E_HP | 5′ GGAAAGCGGTAACAGTGTGGCAGCTTTTGCTGCCACTTGCCCGTATCGGGGTTT 3′ |
| (56) T3R4E_HP | 5′ GTTTGCCACCTCAGAGACCAGGCGTTTTCGCCTGGTTTCCGCCACCGCCAGAAT 3′ |
| (57) T3R6E_HP | 5′ TTATTCATGTCACCAAGCTCGCTGTTTTCAGCGAGCTTTGAAACCATTATTAGC 3′ |

| | |
|---|---|
| (58) T3R8E_HP | 5′ ATACCCAAACACCACGCCTACCGCTTTTGCGGTAGGTTGAATAAGTGACGGAAA 3′ |
| (59) T3R10E_HP | 5′ GCGCATTAATAAGAGCCTGGACGCTTTTGCGTCCAGTTAAGAAACAATAACGGA 3′ |
| (60) T3R12E_CYC_HP | 5′ TGCTCAGTGCCAGTTAGGTGGTCGTTTTCGACCACCTTCAAAATAAACAGGGAA 3′ |
| (61) T5R2F_HP | 5′ AATGCCCCATAAATCCGCTCGGACTTTTGTCCGAGCTTTCATTAAAAGAACCAC 3′ |
| (62) T5R4F_HP | 5′ CACCAGAGTTCGGTCAGCCGAGCGTTTTCGCTCGGCTTTAGCCCCCTCGATAGC 3′ |
| (63) T5R6F_HP | 5′ AGCACCGTAGGGAAGGTCGGAGGCTTTTGCCTCCGATTTAAATATTTTATTTTG 3′ |
| (64) T5R8F_HP | 5′ TCACAATCCCGAGGAACTGGTGGCTTTTGCCACCAGTTACGCAATAATGAAATA 3′ |
| (65) T5R10F_HP | 5′ GCAATAGCAGAGAATACCGCAGGCTTTTGCCTGCGGTTACATAAAAACAGCCAT 3′ |
| (66) T5R12F_CYC_HP | 5′ ATTATTTAGAAGGATTGCCATCGCTTTTGCGATGGCTTAGGATTAGAAACAGTT 3′ |
| (67) T5R2E_HP | 5′ ACAAACAACTGCCTATCACGACGCTTTTGCGTCGTGTTTTCGGAACCTGAGACT 3′ |
| (68) T5R4E_HP | 5′ TCGGCATTCCGCCGCCGTCGCTGCTTTTGCAGCGACTTAGCATTGATGATATTC 3′ |
| (69) T5R6E_HP | 5′ ATTGAGGGAATCAGTACGGAGCACTTTTGTGCTCCGTTGCGACAGACGTTTTCA 3′ |
| (70) T5R8E_HP | 5′ GAAGGAAAAATAGAAAGCCTAGCGTTTTCGCTAGGCTTATTCATATTTCAACCG 3′ |
| (71) T5R10E_HP | 5′ CTTTACAGTATCTTACCGCTCGTGTTTTCACGAGCGTTCGAAGCCCAGTTACCA 3′ |
| (72) T5R12E_CYC_HP | 5′ CCTCAAGATCCCAATCCGTGGAGCTTTTGCTCCACGTTCAAATAAGATAGCAGC 3′ |

**Supplementary Table 3:** DNA origami terminus strands for fluorescent labeling. All labeling strands were ordered unpurified from IDT. Fluorescent strands were ordered HPLC purified from IDT

| Strand Name | Sequence |
|---|---|
| **Fluorescent strands** | |
| atto647 strand | /5ATTO647NN/AAGCGTAGTCGGATCTC 3′ |
| atto488 strand | /5ATTO488N/AAGCGTAGTCGGATCTC 3′ |
| **Labeling strands** | |
| Unused_m13mp18_01 | 5′ AAATTCTTACCAGTATAAAGCCAACTTTTGAGATCCGACTACGC 3′ |
| Unused_m13mp18_02 | 5′ GCCTGTTTAGTATCATATGCGTTATTTTTGAGATCCGACTACGC 3′ |
| Unused_m13mp18_03 | 5′ ACACCGGAATCATAATTACTAGAAATTTTGAGATCCGACTACGC 3′ |
| Unused_m13mp18_04 | 5′ GATAAATAAGGCGTTAAATAAGAATTTTTGAGATCCGACTACGC 3′ |
| Unused_m13mp18_05 | 5′ TTTAATGGTTTGAAATACCGACCGTTTTTGAGATCCGACTACGC 3′ |
| Unused_m13mp18_06 | 5′ TTAGTTAATTTCATCTTCTGACCTATTTTGAGATCCGACTACGC 3′ |
| Unused_m13mp18_07 | 5′ ACGCGAGAAAACTTTTTCAAATATATTTTGAGATCCGACTACGC 3′ |
| Unused_m13mp18_08 | 5′ GATGCAAATCCAATCGCAAGACAAATTTTGAGATCCGACTACGC 3′ |
| Unused_m13mp18_09 | 5′ TGGGTTATATAACTATATGTAAATGTTTTGAGATCCGACTACGC 3′ |
| Unused_m13mp18_10 | 5′ ACTACCTTTTTAACCTCCGGCTTAGTTTTGAGATCCGACTACGC 3′ |
| Unused_m13mp18_11 | 5′ AATTTATCAAAATCATAGGTCTGAGTTTTGAGATCCGACTACGC 3′ |
| Unused_m13mp18_12 | 5′ TTAAGACGCTGAGAAGAGTCAATAGTTTTGAGATCCGACTACGC 3′ |
| Unused_m13mp18_13 | 5′ TCCTTGAAAACATAGCGATAGCTTATTTTGAGATCCGACTACGC 3′ |
| Unused_m13mp18_14 | 5′ TCGCTATTAATTAATTTTCCCTTAGTTTTGAGATCCGACTACGC 3′ |
| Unused_m13mp18_15 | 5′ AGTGAATAACCTTGCTTCTGTAAATTTTTGAGATCCGACTACGC 3′ |
| Unused_m13mp18_16 | 5′ GAAACAGTACATAAATCAATATATGTTTTGAGATCCGACTACGC 3′ |
| Unused_m13mp18_17 | 5′ ATTTCATTTGAATTACCTTTTTTAATTTTGAGATCCGACTACGC 3′ |
| Unused_m13mp18_18 | 5′ AGAAAACAAAATTAATTACATTTAATTTTGAGATCCGACTACGC 3′ |
| Unused_m13mp18_19 | 5′ CAAAAGAAGATGATGAAACAAACATTTTTGAGATCCGACTACGC 3′ |
| Unused_m13mp18_20 | 5′ GCGAATTATTCATTTCAATTACCTGTTTTGAGATCCGACTACGC 3′ |
| Unused_m13mp18_21 | 5′ AATACCAAGTTACAAAATCGCGCAGTTTTGAGATCCGACTACGC 3′ |
| Unused_m13mp18_22 | 5′ CAATAACGGATTCGCCTGATTGCTTTTTTGAGATCCGACTACGC 3′ |
| Unused_m13mp18_23 | 5′ TAACAGTACCTTTTACATCGGGAGATTTTGAGATCCGACTACGC 3′ |
| Unused_m13mp18_24 | 5′ CAGGTTTAACGTCAGATGAATATACTTTTGAGATCCGACTACGC 3′ |
| Unused_m13mp18_25 | 5′ CAGAAATAAAGAAATTGCGTAGATTTTTTGAGATCCGACTACGC 3′ |
| Unused_m13mp18_26 | 5′ CCATATCAAAATTATTTGCACGTAATTTTGAGATCCGACTACGC 3′ |
| Unused_m13mp18_27 | 5′ TCTGAATAATGGAAGGGTTAGAACCTTTTGAGATCCGACTACGC 3′ |
| Unused_m13mp18_28 | 5′ TATAATCCTGATTGTTTGGATTATATTTTGAGATCCGACTACGC 3′ |
| Unused_m13mp18_29 | 5′ GATTATCAGATGATGGCAATTCATCTTTTGAGATCCGACTACGC 3′ |
| Unused_m13mp18_30 | 5′ AAGGAGCGGAATTATCATCATATTCTTTTGAGATCCGACTACGC 3′ |
| Unused_m13mp18_31 | 5′ CATTTTGCGGAACAAAGAAACCACCTTTTGAGATCCGACTACGC 3′ |
| Unused_m13mp18_32 | 5′ TAATTTTAAAAGTTTGAGTAACATTTTTTGAGATCCGACTACGC 3′ |
| Unused_m13mp18_33 | 5′ GTATTAAATCCTTTGCCCGAACGTTTTTTGAGATCCGACTACGC 3′ |
| Unused_m13mp18_34 | 5′ TAGACTTTACAAACAATTCGACAACTTTTGAGATCCGACTACGC 3′ |
| Unused_m13mp18_35 | 5′ ATAATACATTTGAGGATTTAGAAGTTTTTGAGATCCGACTACGC 3′ |
| Unused_m13mp18_36 | 5′ CAACTAATAGATTAGAGCCGTCAATTTTTGAGATCCGACTACGC 3′ |
| Unused_m13mp18_37 | 5′ TATCTAAAATATCTTTAGGAGCACTTTTTGAGATCCGACTACGC 3′ |
| Unused_m13mp18_38 | 5′ ACTGATAGCCCTAAAACATCGCCATTTTTGAGATCCGACTACGC 3′ |

| | |
|---|---|
| Unused_m13mp18_39 | 5′ GAATGGCTATTAGTCTTTAATGCGCTTTTGAGATCCGACTACGC 3′ |
| Unused_m13mp18_40 | 5′ AGAATACGTGGCACAGACAATATTTTTTTGAGATCCGACTACGC 3′ |
| Unused_m13mp18_41 | 5′ ATAGAACCCTTCTGACCTGAAAGCGTTTTGAGATCCGACTACGC 3′ |
| Unused_m13mp18_42 | 5′ ATAAAAGGGACATTCTGGCCAACAGTTTTGAGATCCGACTACGC 3′ |
| Unused_m13mp18_43 | 5′ GCAGATTCACCAGTCACACGACCAGTTTTGAGATCCGACTACGC 3′ |
| Unused_m13mp18_44 | 5′ ATCGTCTGAAATGGATTATTTACATTTTTGAGATCCGACTACGC 3′ |
| Unused_m13mp18_45 | 5′ ATGGAAATACCTACATTTTGACGCTTTTTGAGATCCGACTACGC 3′ |
| Unused_m13mp18_46 | 5′ CCAGCCATTGCAACAGGAAAAACGCTTTTGAGATCCGACTACGC 3′ |
| Unused_m13mp18_47 | 5′ CTGGTAATATCCAGAACAATATTACTTTTGAGATCCGACTACGC 3′ |
| Unused_m13mp18_48 | 5′ GTAGAAGAACTCAAACTATCGGCCTTTTTGAGATCCGACTACGC 3′ |
| Unused_m13mp18_49 | 5′ TGATTAGTAATAACATCACTTGCCTTTTTGAGATCCGACTACGC 3′ |
| Unused_m13mp18_50 | 5′ AAATTAACCGTTGTAGCAATACTTCTTTTGAGATCCGACTACGC 3′ |
| Unused_m13mp18_51 | 5′ CCGAGTAAAAGAGTCTGTCCATCACTTTTGAGATCCGACTACGC 3′ |
| Unused_m13mp18_52 | 5′ GAAGTGTTTTTATAATCAGTGAGGCTTTTGAGATCCGACTACGC 3′ |
| Unused_m13mp18_53 | 5′ GACAGGAACGGTACGCCAGAATCCTTTTTGAGATCCGACTACGC 3′ |
| Unused_m13mp18_54 | 5′ AACAGGAGGCCGATTAAAGGGATTTTTTTGAGATCCGACTACGC 3′ |
| Unused_m13mp18_55 | 5′ TCCTCGTTAGAATCAGAGCGGGAGCTTTTGAGATCCGACTACGC 3′ |
| Unused_m13mp18_56 | 5′ GCTTTGACGAGCACGTATAACGTGCTTTTGAGATCCGACTACGC 3′ |
| Unused_m13mp18_57 | 5′ CGCCGCTACAGGGCGCGTACTATGGTTTTGAGATCCGACTACGC 3′ |
| Unused_m13mp18_58 | 5′ TAACCACCACACCCGCCGCGCTTAATTTTGAGATCCGACTACGC 3′ |
| Unused_m13mp18_59 | 5′ TGGCAAGTGTAGCGGTCACGCTGCGTTTTGAGATCCGACTACGC 3′ |
| Unused_m13mp18_60 | 5′ AAGCGAAAGGAGCGGGCGCTAGGGCTTTTGAGATCCGACTACGC 3′ |
| Unused_m13mp18_61 | 5′ CGAACGTGGCGAGAAAGGAAGGGAATTTTGAGATCCGACTACGC 3′ |
| Unused_m13mp18_62 | 5′ GATTTAGAGCTTGACGGGGAAAGCCTTTTGAGATCCGACTACGC 3′ |
| Unused_m13mp18_63 | 5′ TAAATCGGAACCCTAAAGGGAGCCCTTTTGAGATCCGACTACGC 3′ |
| Unused_m13mp18_64 | 5′ TTTTGGGGTCGAGGTGCCGTAAAGCTTTTGAGATCCGACTACGC 3′ |
| Unused_m13mp18_65 | 5′ TACGTGAACCATCACCCAAATCAAGTTTTGAGATCCGACTACGC 3′ |
| Unused_m13mp18_66 | 5′ AAACCGTCTATCAGGGCGATGGCCCTTTTGAGATCCGACTACGC 3′ |
| Unused_m13mp18_67 | 5′ ACGTGGACTCCAACGTCAAAGGGCGTTTTGAGATCCGACTACGC 3′ |
| Unused_m13mp18_68 | 5′ TTTGGAACAAGAGTCCACTATTAAATTTTGAGATCCGACTACGC 3′ |
| Unused_m13mp18_69 | 5′ CCGAGATAGGGTTGAGTGTTGTTCCTTTTGAGATCCGACTACGC 3′ |
| Unused_m13mp18_70 | 5′ AAATCCCTTATAAATCAAAAGAATATTTTGAGATCCGACTACGC 3′ |
| Unused_m13mp18_71 | 5′ TGTTTGATGGTGGTTCCGAAATCGGTTTTGAGATCCGACTACGC 3′ |
| Unused_m13mp18_72 | 5′ CTGGTTTGCCCCAGCAGGCGAAAATTTTTGAGATCCGACTACGC 3′ |
| Unused_m13mp18_73 | 5′ TGAGAGAGTTGCAGCAAGCGGTCCATTTTGAGATCCGACTACGC 3′ |
| Unused_m13mp18_74 | 5′ AGCTGATTGCCCTTCACCGCCTGGCTTTTGAGATCCGACTACGC 3′ |
| Unused_m13mp18_75 | 5′ TTTCTTTTCACCAGTGAGACGGGCATTTTGAGATCCGACTACGC 3′ |
| Unused_m13mp18_76 | 5′ GTTTGCGTATTGGGCGCCAGGGTGGTTTTGAGATCCGACTACGC 3′ |
| Unused_m13mp18_77 | 5′ GAATCGGCCAACGCGCGGGGAGAGGTTTTGAGATCCGACTACGC 3′ |
| Unused_m13mp18_78 | 5′ GAAACCTGTCGTGCCAGCTGCATTATTTTGAGATCCGACTACGC 3′ |
| Unused_m13mp18_79 | 5′ TGCGCTCACTGCCCGCTTTCCAGTCTTTTGAGATCCGACTACGC 3′ |
| Unused_m13mp18_80 | 5′ GAGTGAGCTAACTCACATTAATTGCTTTTGAGATCCGACTACGC 3′ |
| Unused_m13mp18_81 | 5′ TAAAGTGTAAAGCCTGGGGTGCCTATTTTGAGATCCGACTACGC 3′ |
| Unused_m13mp18_82 | 5′ TTCCACACAACATACGAGCCGGAAGTTTTGAGATCCGACTACGC 3′ |
| Unused_m13mp18_83 | 5′ CTGTGTGAAATTGTTATCCGCTCACTTTTGAGATCCGACTACGC 3′ |
| Unused_m13mp18_84 | 5′ ATTCGTAATCATGGTCATAGCTGTTTTTTGAGATCCGACTACGC 3′ |
| Unused_m13mp18_85 | 5′ TAGAGGATCCCCGGGTACCGAGCTCTTTTGAGATCCGACTACGC 3′ |
| Unused_m13mp18_86 | 5′ CAAGCTTGCATGCCTGCAGGTCGACTTTTGAGATCCGACTACGC 3′ |
| Unused_m13mp18_87 | 5′ ACGACGTTGTAAAACGACGGCCAGTTTTTGAGATCCGACTACGC 3′ |
| Unused_m13mp18_88 | 5′ TTGGGTAACGCCAGGGTTTTCCCAGTTTTGAGATCCGACTACGC 3′ |
| Unused_m13mp18_89 | 5′ AGGGGGATGTGCTGCAAGGCGATTATTTTGAGATCCGACTACGC 3′ |
| Unused_m13mp18_90 | 5′ CTCTTCGCTATTACGCCAGCTGGCGTTTTGAGATCCGACTACGC 3′ |
| Unused_m13mp18_91 | 5′ CTGTTGGGAAGGGCGATCGGTGCGGTTTTGAGATCCGACTACGC 3′ |
| Unused_m13mp18_92 | 5′ GCGCCATTCGCCATTCAGGCTGCGCTTTTGAGATCCGACTACGC 3′ |
| Unused_m13mp18_93 | 5′ CGCTTCTGGTGCCGGAAACCAGGCATTTTGAGATCCGACTACGC 3′ |
| Unused_m13mp18_94 | 5′ ATCGCACTCCAGCCAGCTTTCCGGCTTTTGAGATCCGACTACGC 3′ |
| Unused_m13mp18_95 | 5′ GACGACGACAGTATCGGCCTCAGGATTTTGAGATCCGACTACGC 3′ |
| Unused_m13mp18_96 | 5′ GTAACCGTGCATCTGCCAGTTTGAGTTTTGAGATCCGACTACGC 3′ |
| Unused_m13mp18_97 | 5′ GGTCACGTTGGTGTAGATGGGCGCATTTTGAGATCCGACTACGC 3′ |
| Unused_m13mp18_98 | 5′ AAACGGCGGATTGACCGTAATGGGATTTTGAGATCCGACTACGC 3′ |
| Unused_m13mp18_99 | 5′ ACAACCCGTCGGATTCTCCGTGGGATTTTGAGATCCGACTACGC 3′ |
| Unused_m13mp18_100 | 5′ TTCATCAACATTAAATGTGAGCGAGTTTTGAGATCCGACTACGC 3′ |

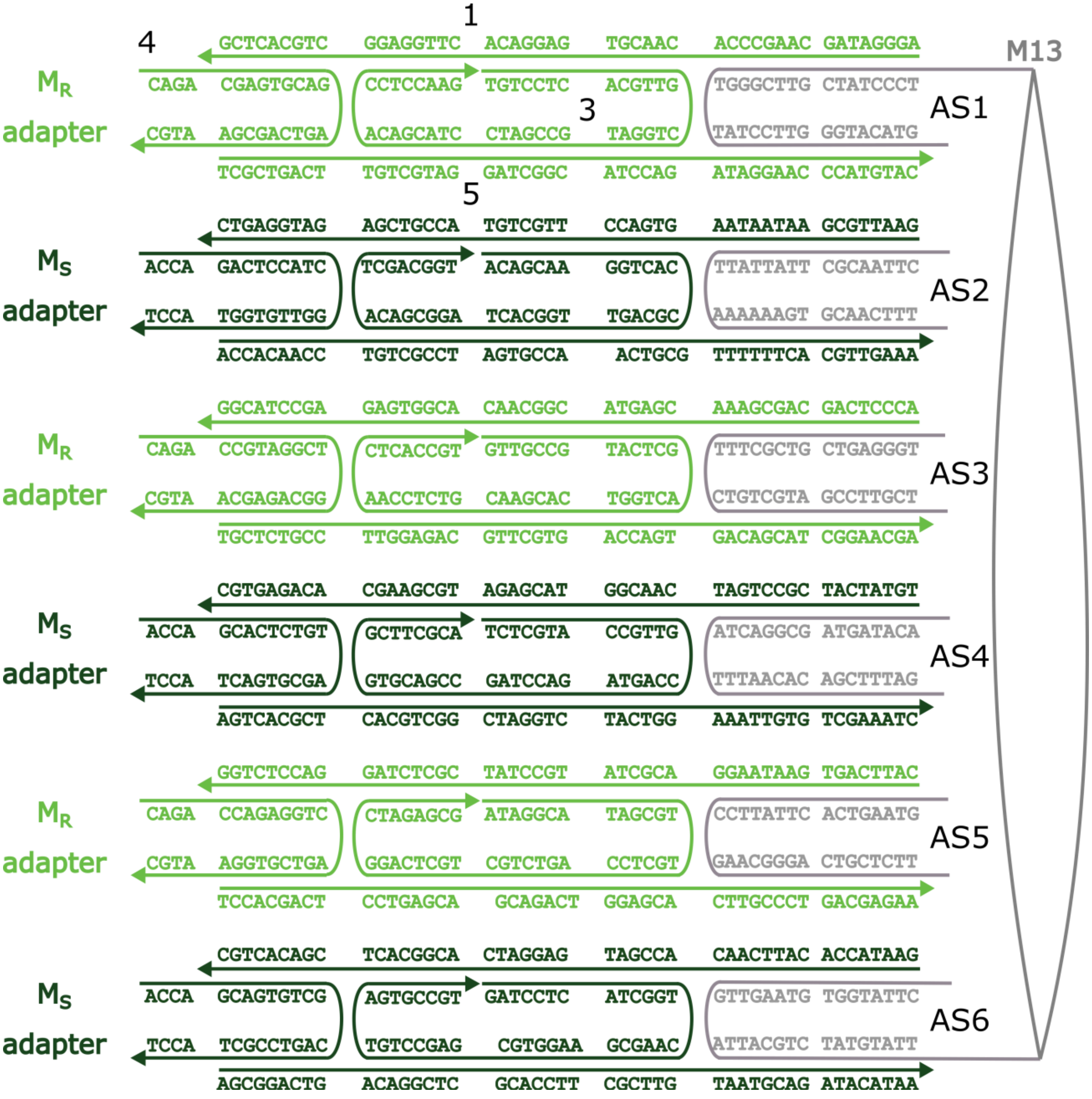


**Supplementary Figure 3:** DNA origami seed adapter design. There are 6 monomer adapters around the circumference of the seed face (AS1 – AS6), 3 adapters that present the $M_R$ sticky ends and 3 adapters that present the $M_S$ sticky ends. The adapters resemble DNA monomers but are bound to M13 DNA (gray) where strand 2 would normally be on the monomers (Supplementary Note 1).

**Supplementary Table 4:** Sequences of DNA origami seed monomer adapters. All strands were ordered from Integrated DNA Technologies, Inc (IDT). Strands 1, 3, and 5 were all ordered unpurified and all sticky end strands (strand 4) were ordered PAGE purified.

| **Strand name** | **Sequence** |
|---|---|
| $M_R$ S adapter AS1-1 | 5′ AGGGATAGCAAGCCCACAACGTGAGGACACTTGGAGGCTGCACTCG 3′ |
| $M_R$ S adapter AS1-3 | 5′ TGTCCTCACGTTGCTGGATGCCGATCCTACGACACCTCCAAG 3′ |
| $M_R$ S adapter AS1-5 | 5′ TCGCTGACTTGTCGTAGGATCGGCATCCAGATAGGAACCCATGTAC 3′ |
| $M_R$ S adapter AS1-4 | 5′ CAGACGAGTGCAGAGTCAGCGAATGC 3′ |
| | |
| $M_S$ S adapter AS2-1 | 5′ GAATTGCGAATAATAAGTGACCTTGCTGTACCGTCGAGATGGAGTC 3′ |
| $M_S$ S adapter AS2-3 | 5′ ACAGCAAGGTCACCGCAGTTGGCACTAGGCGACATCGACGGT 3′ |
| $M_S$ S adapter AS2-5 | 5′ ACCACAACCTGTCGCCTAGTGCCAACTGCGTTTTTTCACGTTGAAA 3′ |
| $M_S$ S adapter AS2-4 | 5′ ACCAGACTCCATCGGTTGTGGTACCT 3′ |
| | |
| $M_R$ S adapter AS3-1 | 5′ ACCCTCAGCAGCGAAACGAGTACGGCAACACGGTGAGAGCCTACGG 3′ |
| $M_R$ S adapter AS3-3 | 5′ GTTGCCGTACTCGACTGGTCACGAACGTCTCCAACTCACCGT 3′ |
| $M_R$ S adapter AS3-5 | 5′ TGCTCTGCCTTGGAGACGTTCGTGACCAGTGACAGCATCGGAACGA 3′ |
| $M_R$ S adapter AS3-4 | 5′ CAGACCGTAGGCTGGCAGAGCAATGC 3′ |
| | |
| $M_S$ S adapter AS4-1 | 5′ TGTATCATCGCCTGATCAACGGTACGAGATGCGAAGCACAGAGTGC 3′ |
| $M_S$ S adapter AS4-3 | 5′ TCTCGTACCGTTGCCAGTAGACCTAGCCGACGTGGCTTCGCA 3′ |
| $M_S$ S adapter AS4-5 | 5′ AGTCACGCTCACGTCGGCTAGGTCTACTGGAAATTGTGTCGAAATC 3′ |
| $M_S$ S adapter AS4-4 | 5′ ACCAGCACTCTGTAGCGTGACTACCT 3′ |
| | |
| $M_R$ S adapter AS5-1 | 5′ CATTCAGTGAATAAGGACGCTATGCCTATCGCTCTAGGACCTCTGG 3′ |
| $M_R$ S adapter AS5-3 | 5′ ATAGGCATAGCGTTGCTCCAGTCTGCTGCTCAGGCTAGAGCG 3′ |
| $M_R$ S adapter AS5-5 | 5′ TCCACGACTCCTGAGCAGCAGACTGGAGCACTTGCCCTGACGAGAA 3′ |
| $M_R$ S adapter AS5-4 | 5′ CAGACCAGAGGTCAGTCGTGGAATGC 3′ |
| | |
| $M_S$ S adapter AS6-1 | 5′ GAATACCACATTCAACACCGATGAGGATCACGGCACTCGACACTGC 3′ |
| $M_S$ S adapter AS6-3 | 5′ GATCCTCATCGGTCAAGCGAAGGTGCGAGCCTGTAGTGCCGT 3′ |
| $M_S$ S adapter AS6-5 | 5′ AGCGGACTGACAGGCTCGCACCTTCGCTTGTAATGCAGATACATAA 3′ |
| $M_S$ S adapter AS6-4 | 5′ ACCAGCAGTGTCGCAGTCCGCTACCT 3′ |

**Supplementary Note 3: DNA nanostructured cap design and sequences**

The design of the nanostructured cap was adopted from previous studies[3]. A cap is composed of a scaffold strand (M13mp18 DNA (7,240 bases) purchased from New England Biolabs) and 24 adapter strands (strands on the adapters possess the monomer sticky end sequences). Unlike the seed, the cap does not possess any staple strands. The resulting assembly is unstructured, which creates an entropic barrier to the cap's nucleating any new growth[3]. The labeling scheme for the cap was the same as the seeds other than the fluorescent strand was modified with atto647 (sequences are in Supplementary Table 3). The sequences of the adapters are presented in Supplementary Table 5.

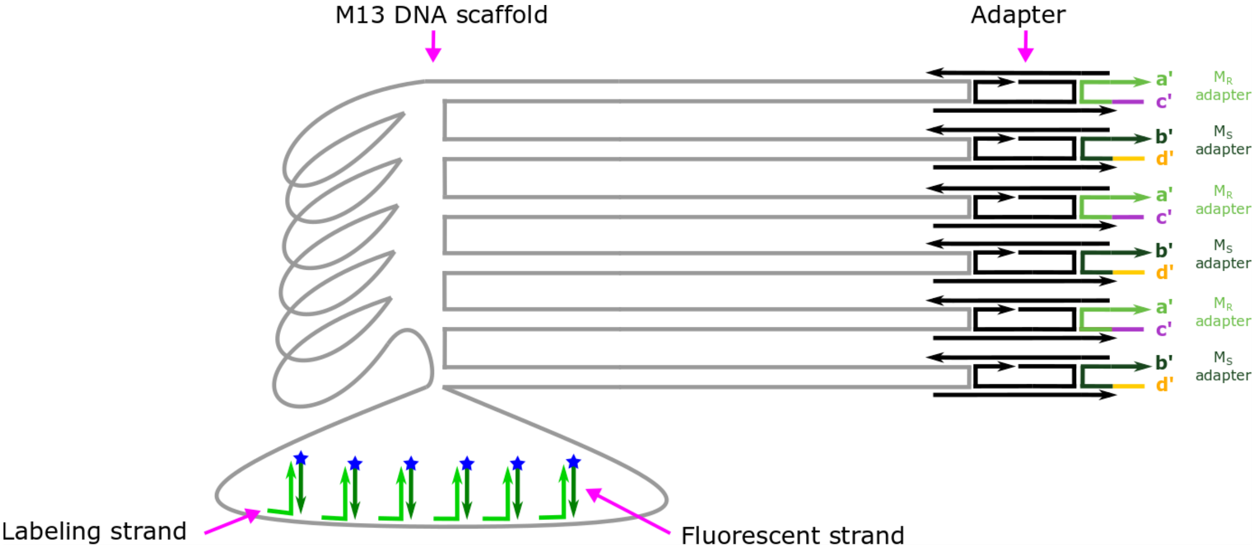


**Supplementary Figure 4:** DNA origami cap design.

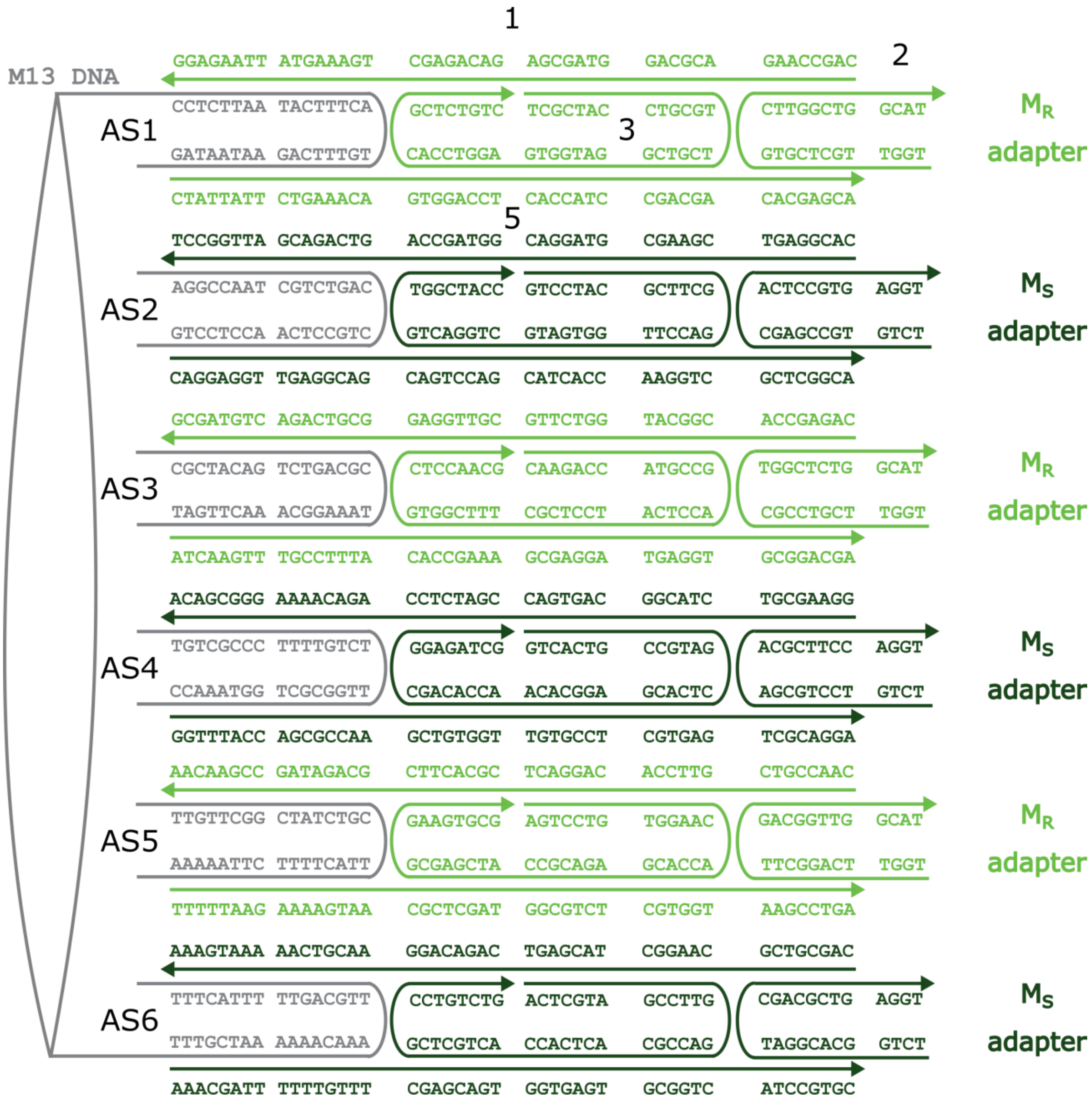


**Supplementary Figure 5:** DNA origami cap adapter design. There are 6 monomer adapters on the cap (AS1 – AS6), 3 adapters that present the $M_R$ sticky ends and 3 adapters that present the $M_S$ sticky ends. The adapters resemble DNA monomers composed but are bound to M13 DNA (gray) where strand 4 would normally be on the monomers (Supplementary Note 1).

**Supplementary Table 5:** Sequences of DNA cap monomer adapters. All strands were ordered from Integrated DNA Technologies, Inc (IDT). Strands 1, 3, and 5 were all ordered unpurified and all sticky end strands (strand 2) were ordered PAGE purified.

| **Strand name** | **Sequence** |
|---|---|
| $M_R$ C adapter AS1-1 | 5′ CAGCCAAGACGCAGGTAGCGAGACAGAGCTGAAAGTATTAAGAGG 3′ |
| $M_R$ C adapter AS1-3 | 5′ TCGCTACCTGCGTTCGTCGGATGGTGAGGTCCACGCTCTGTC 3′ |
| $M_R$ C adapter AS1-5 | 5′ CTATTATTCTGAAACAGTGGACCTCACCATCCGACGACACGAGCA 3′ |
| $M_R$ C adapter AS1-2 | 5′ TGGTTGCTCGTGCTTGGCTGGCAT 3′ |
| | |
| $M_S$ C adapter AS2-1 | 5′ CACGGAGTCGAAGCGTAGGACGGTAGCCAGTCAGACGATTGGCCT 3′ |
| $M_S$ C adapter AS2-3 | 5′ GTCCTACGCTTCGGACCTTGGTGATGCTGGACTGTGGCTACC 3′ |
| $M_S$ C adapter AS2-5 | 5′ CAGGAGGTTGAGGCAGCAGTCCAGCATCACCAAGGTCGCTCGGCA 3′ |
| $M_S$ C adapter AS2-2 | 5′ TCTGTGCCGAGCACTCCGTGAGGT 3′ |
| | |
| $M_R$ C adapter AS3-1 | 5′ CAGAGCCACGGCATGGTCTTGCGTTGGAGGCGTCAGACTGTAGCG 3′ |
| $M_R$ C adapter AS3-3 | 5′ CAAGACCATGCCGACCTCATCCTCGCTTTCGGTGCTCCAACG 3′ |
| $M_R$ C adapter AS3-5 | 5′ ATCAAGTTTGCCTTTACACCGAAAGCGAGGATGAGGTGCGGACGA 3′ |
| $M_R$ C adapter AS3-2 | 5′ TGGTTCGTCCGCTGGCTCTGGCAT 3′ |
| | |
| $M_S$ C adapter AS4-1 | 5′ GGAAGCGTCTACGGCAGTGACCGATCTCCAGACAAAAGGGCGACA 3′ |
| $M_S$ C adapter AS4-3 | 5′ GTCACTGCCGTAGCTCACGAGGCACAACCACAGCGGAGATCG 3′ |
| $M_S$ C adapter AS4-5 | 5′ GGTTTACCAGCGCCAAGCTGTGGTTGTGCCTCGTGAGTCGCAGGA 3′ |
| $M_S$ C adapter AS4-2 | 5′ TCTGTCCTGCGAACGCTTCCAGGT 3′ |
| | |
| $M_R$ C adapter AS5-1 | 5′ CAACCGTCGTTCCACAGGACTCGCACTTCGCAGATAGCCGAACAA 3′ |
| $M_R$ C adapter AS5-3 | 5′ AGTCCTGTGGAACACCACGAGACGCCATCGAGCGGAAGTGCG 3′ |
| $M_R$ C adapter AS5-5 | 5′ TTTTTAAGAAAAGTAACGCTCGATGGCGTCTCGTGGTAAGCCTGA 3′ |
| $M_R$ C adapter AS5-2 | 5′ TGGTTCAGGCTTGACGGTTGGCAT 3′ |
| | |
| $M_S$ C adapter AS6-1 | 5′ CAGCGTCGCAAGGCTACGAGTCAGACAGGAACGTCAAAAATGAAA 3′ |
| $M_S$ C adapter AS6-3 | 5′ ACTCGTAGCCTTGGACCGCACTCACCACTGCTCGCCTGTCTG 3′ |
| $M_S$ C adapter AS6-5 | 5′ AAACGATTTTTTGTTTCGAGCAGTGGTGAGTGCGGTCATCCGTGC 3′ |
| $M_S$ C adapter AS6-2 | 5′ TCTGGCACGGATCGACGCTGAGGT 3′ |

**Supplementary Note 4: Modeling unregulated seeded nanotube growth with monomer depletion**

To model seeded nanotube growth, we used a stochastic kinetic model of monomer attachment and detachment similar to models presented previously[1,4]. We used the Gillespie algorithm[5] to simulate the growth of a population of nanotubes six monomers in circumference from stable nucleating facets *(i.e.* seeds). Monomers could only attach to a growth face if the attachment formed at least two bonds between the monomer and the seed or nanotube facet. The simulation allowed reversible binding of monomers to nanotube growth faces or seeds (Supplementary Fig. 6). The model has three free parameters: the rate constant for monomers attaching to a seed and/or a growing nanotube face ($k_{ON}$), the off rate of a monomer attached to a nanotube growth face ($k_{OFF,\ M\text{-}NT}$), and the off rate of a monomer attached to a seed ($k_{OFF,\ M\text{-}S}$).

$k_{ON}$ has been measured for DNA monomers similar to those used in this study under similar conditions[4] to be between $10^5 – 10^6\ M^{-1}s^{-1}$. The rate at which a monomer detaches from a nanotube or seed depends on the strength of the hybridization interaction ($\Delta G^o{}_i < 0$) through Supplementary Equation 1, where *i* refers to either the monomer-seed or monomer-nanotube interaction, R refers to the gas constant, and T refers to the absolute temperature.

$$(1)\ \ k_{OFF,i} = k_{ON} \exp\left(\frac{\Delta G_i^o}{RT}\right)$$

A nucleation barrier for growth from the origami seed was included in the model because its inclusion in models was previously found to explain the broadly distributed length of nanotubes that formed during a seeded growth process[1]. The existence of the nucleation barrier was modeled, as in previous work, by assuming an off rate for monomers attached to the seed $k_{OFF,\ M\text{-}S}$ that is higher than the off rate of monomers attached to an existing nanotube growth face, *i.e.* $k_{OFF,\ M\text{-}S} > k_{OFF,\ M\text{-}NT}$.

For most simulations, growth of nanotubes from 250 seeds was tracked. An initial monomer concentration of 150 nM was used in the simulations. The seed concentration in the simulations was set (by setting the simulation volume) to be the viable seed concentrations used in our experiments *i.e.* 75% of the 0.1 nM, 0.33 nM, or 1 nM seeds actually added to experimental samples (Supplementary Note 6).

To find model parameters that closely recapitulated our experimental results for growth using 150 nM monomers, we conducted a series of parameter sweep simulations with $k_{ON}$ spanning from $10^5 – 10^6\ M^{-1}s^{-1}$, $\Delta G^o{}_{M\text{-}NT}$ spanning from -9.0 to - 10 kcal/mol, and $k_{OFF,\ M\text{-}S}$ spanning 5* $k_{OFF,\ M\text{-}NT}$ to 25* $k_{OFF,\ M\text{-}NT}$. From these simulations we found that the values: $k_{ON} = 2\text{x}10^5\ M^{-1}s^{-1}$, $k_{OFF,\ M\text{-}NT} = 0.0231\ s^{-1}$ (corresponding to a $\Delta G^o{}_{M\text{-}NT}$ = -9.3 kcal/mol), and $k_{OFF,\ M\text{-}S}$ = 6* $k_{OFF,\ M\text{-}NT}$ closely recapitulated our experimental data (Supplementary Fig. 7). Unless otherwise stated, these parameters were used in all subsequent modeling of nanotube growth kinetics in this study.

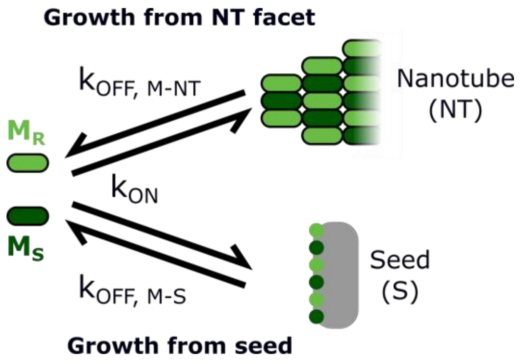


**Supplementary Figure 6:** Reactions of the stochastic kinetic model for unregulated growth. Monomers can reversibly bind to a growing nanotube or to seed face where a monomer can bind *via* two sticky ends. The presence of a nucleation barrier for the seed[1] was modeled as a higher off rate for monomers bound to a seed than monomers bound to a nanotube.

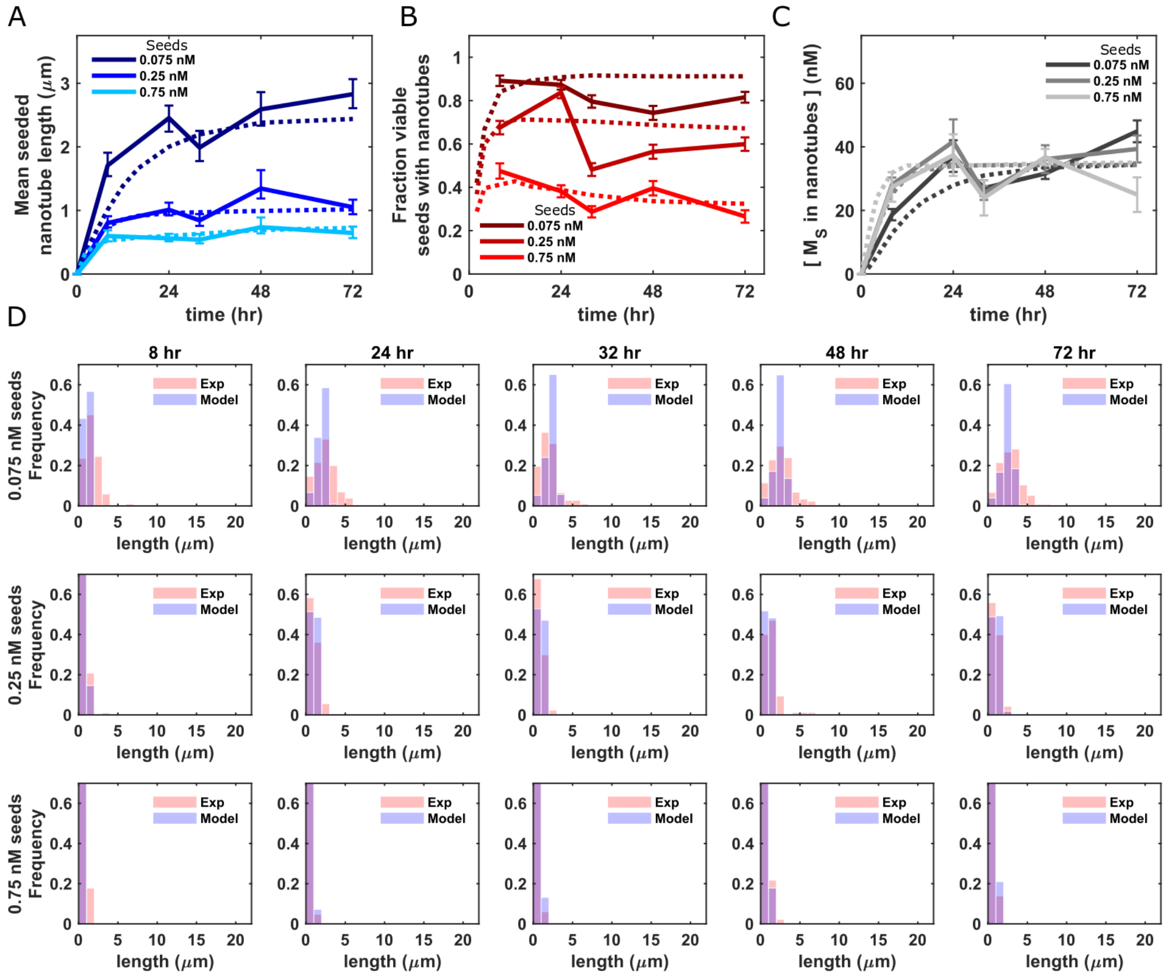


**Supplementary Figure 7:** A stochastic kinetic model recapitulates experimentally measured nanotube growth observables over time during nanotube growth with 150 nM monomers (Fig. 2 of the main text). (**A-D**) Comparison of experimental measurements and stochastic simulation predictions of mean seeded nanotube lengths (A), fractions of viable seeds with nanotubes (B), concentration of monomers incorporated into nanotubes (C), and nanotube length distributions (D) during unregulated nanotube growth with 150 nM monomers. In panels A-C: dashed lines represent simulation results, solid lines experimental measurements. In (A) and (B), error bars represent 95% confidence intervals and in (C), error bars represent standard deviation across images. Coefficient of variation values for the histograms are in Supplementary Table 6. Stochastic simulations were conducted using: $k_{ON} = 2\times10^5$ $M^{-1}s^{-1}$, $k_{OFF,\ M\text{-}NT} = 0.0231$ $s^{-1}$, $k_{OFF,\ M\text{-}S} = 6 * k_{OFF,\ M\text{-}NT}$.

**Supplementary Table 6:** Dispersity of nanotube length distributions computed as the coefficient of variation (CV) for unregulated growth with 150 nM monomers. CV values computed from the experimental and simulation results in Supplementary Figure 7 and are presented as percentages.

| | 0.075 nM seeds | | 0.25 nM seeds | | 0.75 nM seeds | |
|---|---|---|---|---|---|---|
| | **Exp** | **Model** | **Exp** | **Model** | **Exp** | **Model** |
| **8 hr** | 54% | 30% | 54% | 32% | 50% | 37% |
| **24 hr** | 49% | 23% | 53% | 35% | 40% | 43% |
| **32 hr** | 62% | 25% | 53% | 35% | 47% | 46% |
| **48 hr** | 50% | 26% | 73% | 39% | 55% | 45% |
| **72 hr** | 48% | 25% | 53% | 45% | 44% | 50% |

## Supplementary Note 5: Modeling unregulated seeded nanotube growth without monomer depletion

To model the nanotube growth process without monomer depletion, the stochastic model as presented in Supplementary Note 4 with the same nanotube growth parameters as was used: $k_{ON}$ = 2x10$^5$ M$^{-1}$s$^{-1}$, $k_{OFF,\ M\text{-}NT}$ = 0.0231 s$^{-1}$, and $k_{OFF,\ M\text{-}S}$ = 6* $k_{OFF,\ M\text{-}NT}$. The number of monomers in the simulation was set so that the concentration of monomers would be 150 nM for the volume simulated. In these simulations, however, attachment to or detachment of monomers from nanotubes or seeds did not change the number of free monomers (and thus their concentration) present in the simulation volume.

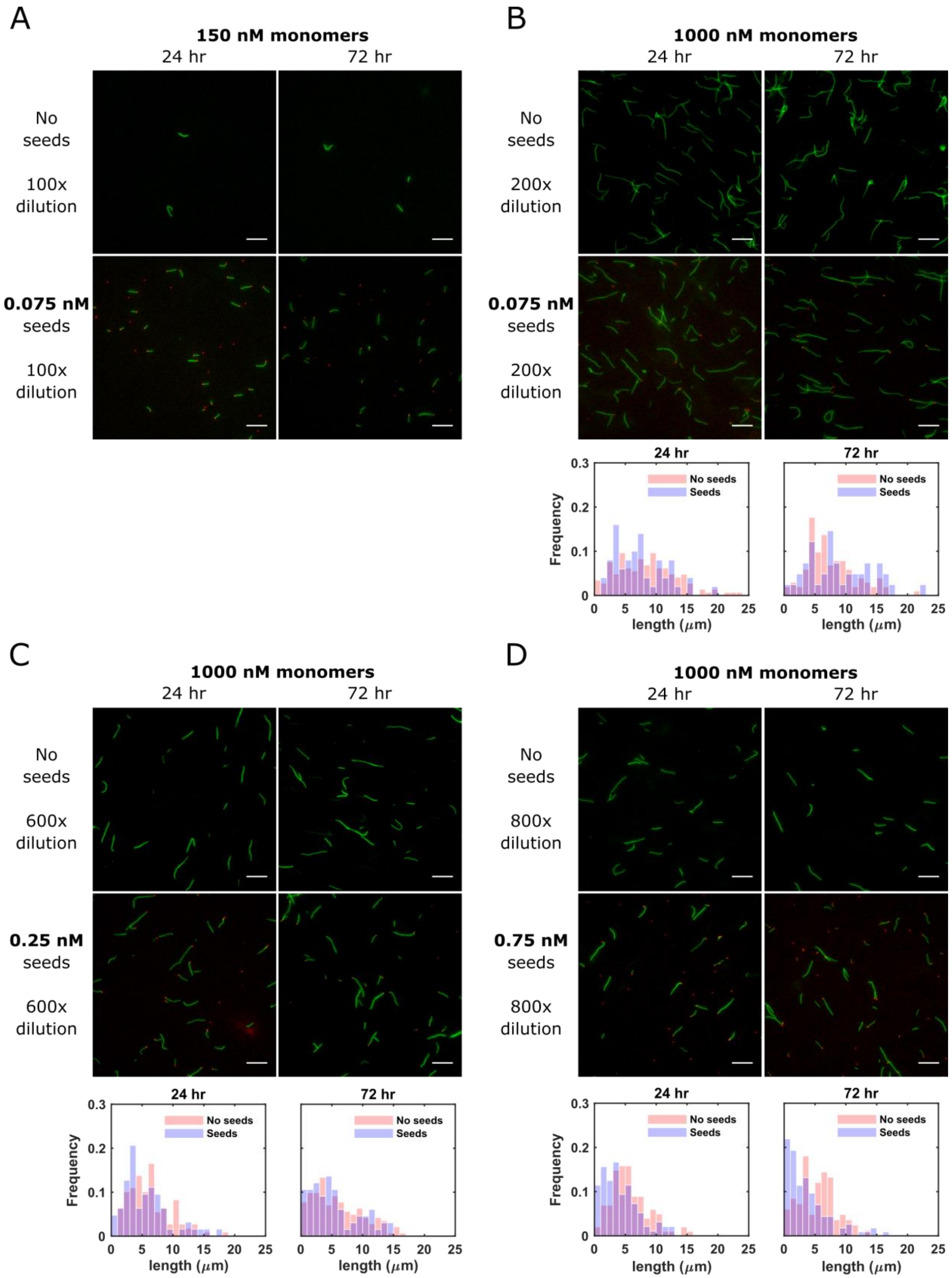
A
150 nM monomers
24 hr
72 hr
No seeds
100x dilution
0.075 nM seeds
100x dilution
B
1000 nM monomers
24 hr
72 hr
No seeds
200x dilution
0.075 nM seeds
200x dilution
No seeds
Seeds
Frequency
length (μm)
C
1000 nM monomers
24 hr
72 hr
No seeds
600x dilution
0.25 nM seeds
600x dilution
D
1000 nM monomers
24 hr
72 hr
No seeds
800x dilution
0.75 nM seeds
800x dilution

E

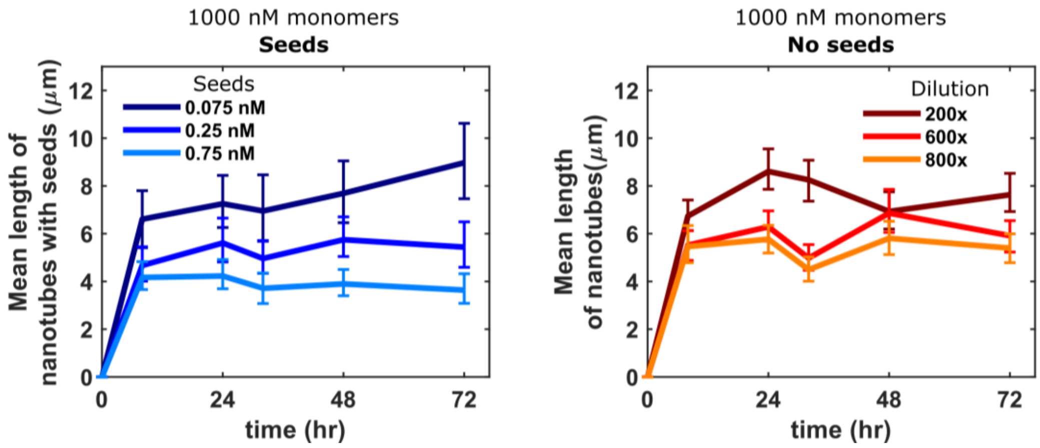


**Supplementary Figure 8:** Unregulated growth with 150 nM or 1000 nM monomers and with and without seeds. (**A**) Fluorescence micrographs of nanotubes grown using 150 nM monomers without and with 0.075 nM viable seeds (Supplementary Note 6). The lack of nanotube growth when seeds are absent in the reaction indicates that growth with 150 nM monomers requires seeds. The monomer concentration of 150 nM is therefore within the seeded growth regime. (**B**,**C**,**D**) Fluorescence micrographs of nanotubes grown using 1000 nM monomers without and with different seed concentrations. Scale bars: 10 µm. For appropriate comparison to the samples with seeds, samples without seeds were imaged at the same dilutions as corresponding samples with seeds. The distributions of nanotube lengths are shown below the fluorescence micrographs. (**E**) Mean nanotube lengths for nanotubes grown using 1000 nM monomers with (left) and without (right) seeds after different durations of growth. For samples with seeds, only the nanotubes with seeds were used to compute nanotube lengths. Nanotubes grown using 1000 nM monomers with and without seeds produce similar numbers of nanotubes (B-D, micrographs), similar length distributions at the times where these distributions were characterized (B-D, histograms), and similar mean lengths (E). Error bars represent 95% confidence intervals from bootstrapping. These similarities suggest that the seeds do not direct the growth process in this monomer concentration regime and suggest that many nanotubes with seeds are likely the product of seeds binding to nanotubes that nucleated and grew spontaneously.

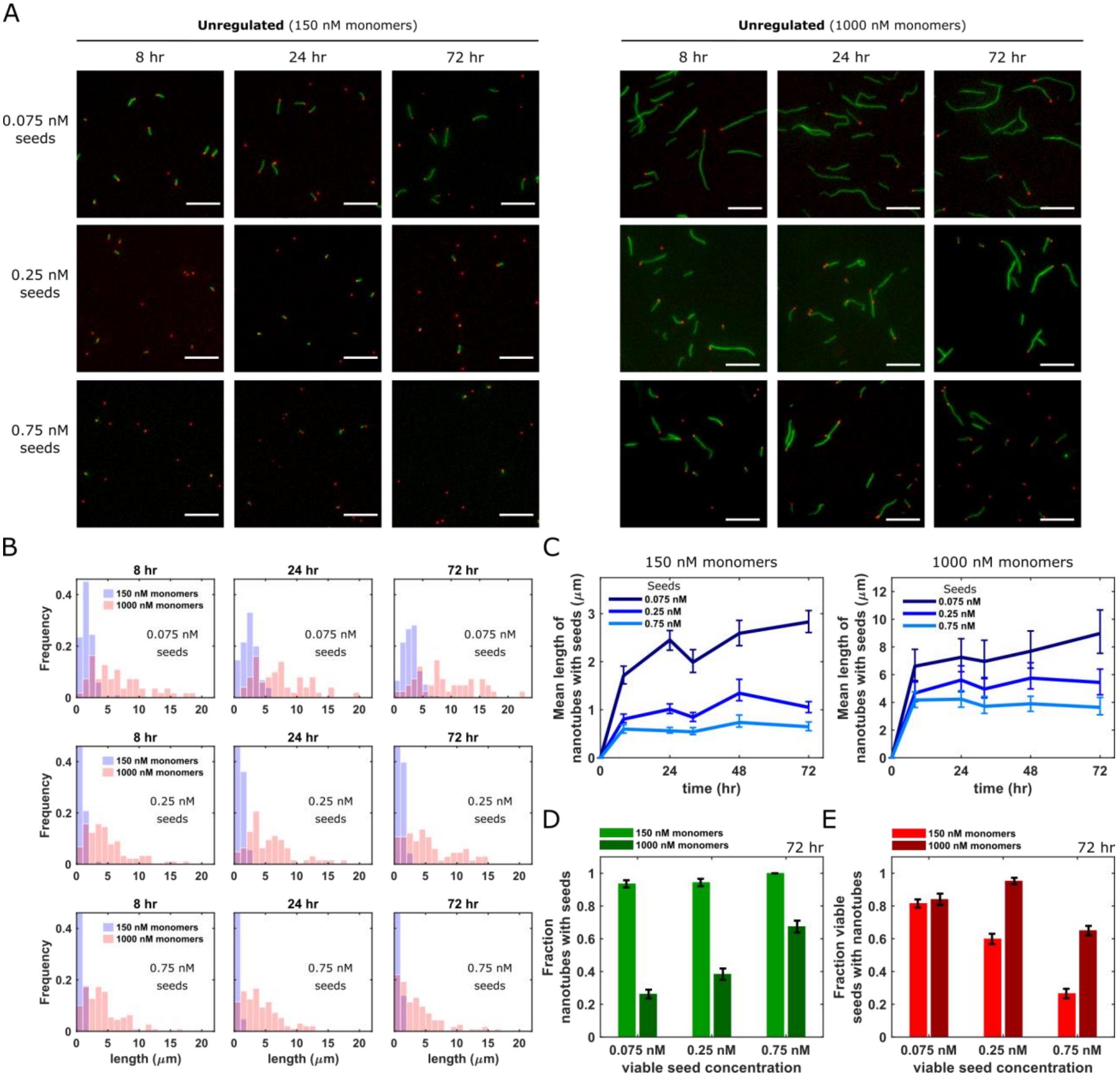


**Supplementary Figure 9:** Characterization of unregulated DNA nanotube growth using 150 nM or 1000 nM monomers. (**A**) Fluorescence micrographs of nanotubes and seeds during growth with the specified monomer and seed concentrations. Scale bars: 10 µm. (**B**) Histograms of seeded nanotube lengths for the samples in (A). (**C**) Mean lengths of nanotubes with seeds during growth with different seed concentrations. Error bars represent 95% confidence intervals from bootstrapping. (**D**,**E**) Fractions of nanotubes with seeds (D) and fractions of viable seeds with nanotubes (E) after 72 hours of growth as a function of seed concentration for two different monomer concentrations. Error bars represent 95% confidence intervals of proportions. The sample sizes for every timepoint of each sample are tabulated in Supplementary Note 14.

**Supplementary Note 6: Determining concentration of viable seeds**

We observed that in an unregulated nanotube growth process using 150 nM monomers, only roughly 65-70% of the seeds nucleated growth even at the lowest seed concentrations tested (Supplementary Fig. 10A, top). We initially assumed this fraction being smaller than 1 was just the inherent yield of the seeds for the growth conditions we selected (*i.e.* it is possible to nucleate nearly 100% of the seeds but under these growth conditions we were not getting the maximum possible nucleation yield). However, our simulations of buffer-regulated nanotube growth (Fig. 4 of the main text) predicted that monomer buffering should increase the fraction of seeds nucleating nanotubes to 1 for all of the seed concentrations we tested experimentally. Our experiments resulted in only 70 – 80% of the seeds nucleating nanotubes across *all* the seed concentrations (Supplementary Fig. 10A, bottom). Our kinetic model of buffer-regulated nanotube growth (Fig. 4 of the main text), which included a small energy barrier to nucleation of nanotubes from seeds, cannot recapitulate these results because the fraction of seeds that nucleate nanotubes has to increase as seed concentration decreases. (*i.e.* we would expect the same trend observed in Supplementary Fig. 10A, top). In other words, the only way our model can predict the same percentage of seeds nucleating nanotubes for all seed concentrations is if all of the seeds nucleate nanotubes.

One potential reason for some seeds being unable to nucleate nanotubes would be if the seeds were not properly formed. The use of adapter strands that were simply synthesized and desalted rather than being further purified could lead to seeds presenting an incomplete set of binding sites for nanotube growth, which could increase the effective barrier to nucleation, or the seeds themselves could be poorly formed[6]. Notably, maximum seed yields of roughly 70 - 80% are consistent with previous results where even with very low seed concentrations (10 pM), only 74% of seeds similar to those used here nucleated nanotubes.

The results of the experiment presented in Figure 6A-C of the main text also support the idea that some seeds have a much higher barrier to nucleation or cannot serve as templates because they are malformed. In this experiment, seeds with one fluorescent dye (S1) were initially incubated with the monomer buffering species for 24 hours, during which time roughly 75-80% of these seeds nucleated nanotubes. After this 24 hours of growth, seeds identical to the S1 seeds but labeled with a different fluorescent dye (S2) were added. By the end of the experiment 75-80% of the S2 seeds had nucleated nanotubes while the fraction of S1 seeds nucleating nanotubes remained the same (Supplementary Fig. 10B). These results indicate that the S1 seeds that did not nucleate nanotubes within the first 24 hours of growth were less able to nucleate nanotubes than the S1 seeds that did nucleate nanotubes: when the S2 seeds were added, these seeds, which are identical to S1 seeds except for the fluorescent label, were able to nucleate nanotubes, while the remaining S1 seeds did not do so. These results thus suggest that the *viable* concentration, *i.e.* the concentration of seeds that are able to nucleate nanotubes within the Nucleation and Growth regime (II) is only 70-80% of the seeds that are detectable *via* fluorescence. As a result of these observations, we assumed that only 75% of the seeds added were capable of readily nucleating nanotubes. The concentration of these seeds, which we termed viable seeds, was assumed to be 75% of the concentration of added seeds and was used to characterize the fractions of seeds that nucleate nanotubes and as the concentration of seeds in stochastic simulations (Supplementary Fig. 10).

As seen in Supplementary Figure 10, the fraction of seeds that nucleated nanotubes varied somewhat from experiment to experiment as well as across timepoints in the same experiment. For example, in

the experiment presented in the bottom panel of Supplementary Figure 10A, the fraction of seeds that nucleated nanotubes was close to 0.75 for the first three timepoints and then drops to a little less than 0.7 for the last two timepoints. These variations suggest that the percentage of viable seeds is likely between 70-75%. We opted to assume 75% for all experiments and timepoints as that ensured that the fraction of viable seeds with nanotubes would rarely exceed 1 for all of our experimental observations.

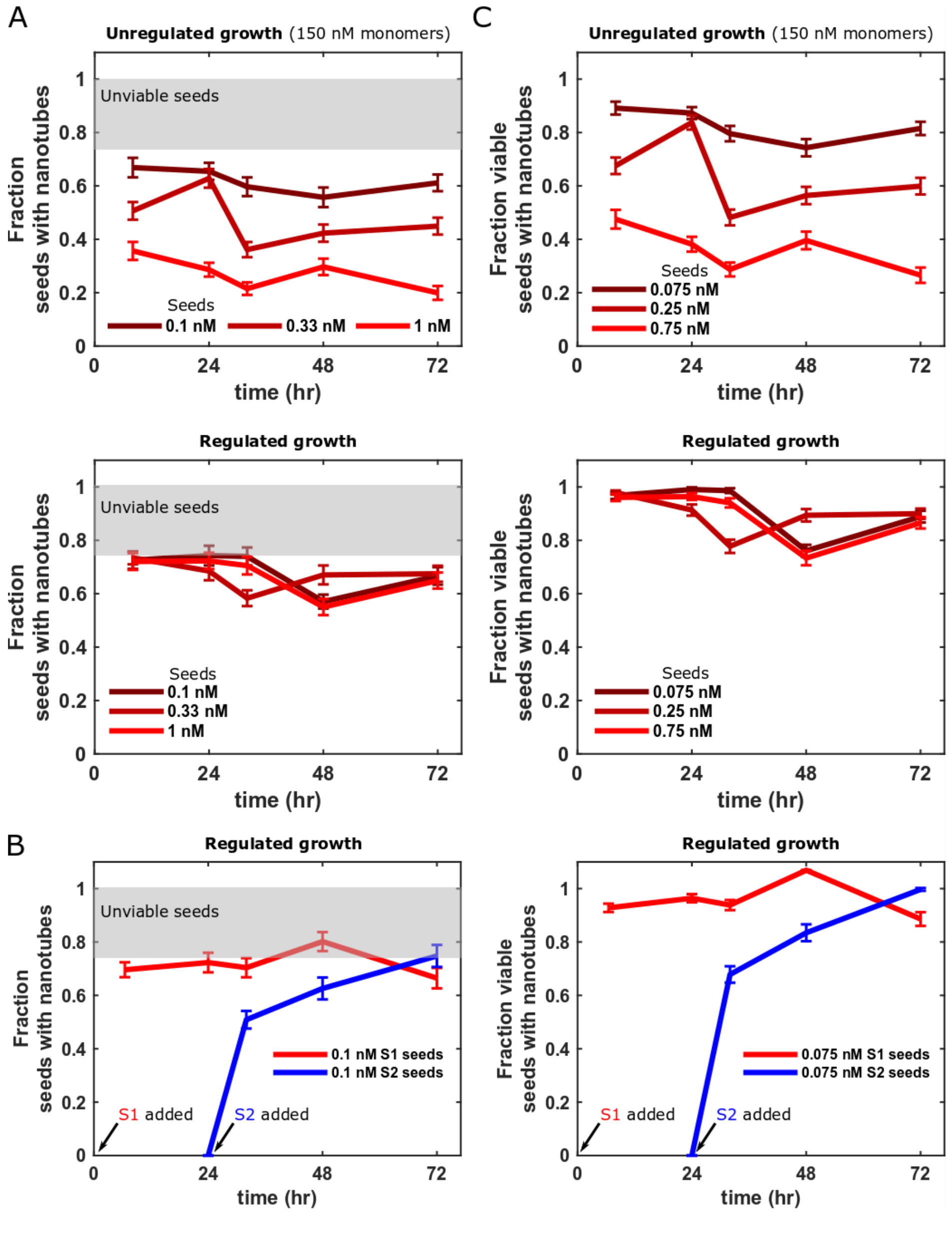


**Supplementary Figure 10:** The concentration of viable seeds is distinct from the concentration of seeds added, as some seeds cannot nucleate nanotubes. (**A**) Fractions of seeds (using the concentration as added) with nanotubes during unregulated growth with 150 nM monomers (top) or regulated growth (bottom) at different seed concentrations. These results are from the experiments presented in Figure 2 and Figure 5 of the main text, respectively. (**B**) Fractions of both seed types (using the concentration as added) with nanotubes during buffer-regulated growth from S1 and S2 seeds; S2 seeds were added after 24 hours. These results are from the experiment presented in Figure 6 of the main text. (**C**) The data from (A) and (B) given as the fractions of viable seeds normalized by the concentration of viable seeds that can nucleate nanotubes. The viable seed concentration was 75% of the total concentration; the remainder of the seeds presented too high of a nucleation barrier to allow for nanotube nucleation. Error bars represent 95% confidence intervals of proportions.

**Supplementary Note 7: Determination of the ranges of monomer concentrations corresponding to regimes II (Seeded Nucleation & Growth), III (Growth only) and IV (No Growth)**

Nanotube growth should cease when the monomer ON and OFF rates are equal. The ON rate is defined as $k_{ON}$*[Free monomers]*[attachment sites] and the OFF rate is defined as $k_{OFF,M\text{-}NT}$*[detachment sites] so the [Free monomers] where the ON and OFF rates are equal is defined as $k_{OFF,M\text{-}NT}$ / $k_{ON}$. Using the rate constants determined from our simulations (Supplementary Note 4) we found the monomer concentration where growth ceases (regime IV, No growth) to be 115 nM.

Because the off rate of monomers from seeds is higher than the off rate of monomers from nanotubes in our model, there should be a range of monomer concentrations where nucleation of new nanotubes from seeds is not favorable but nanotube growth is still favorable. Simulations indicate that the presence of this monomer concentration regime where nanotube growth can occur but additional nucleation from seeds is unfavorable explains the dependence of nucleation yield on seed concentration *i.e.* higher seed concentrations result in lower nucleation yields (Supplementary Fig. 11A-C).

To identify the monomer concentration that marks the boundary between the seeded nucleation and growth and the growth only regime, we conducted stochastic kinetic simulations of nanotube growth *without* monomer depletion with different initial monomer concentrations and looked at seed nucleation as a function of time (Supplementary Fig. 11D). By plotting the initial seed nucleation rates (initial slopes of the simulation data in Supplementary Fig. 11D) as a function of initial monomer concentration, we found that in simulation, at monomer concentrations below 125 nM, fewer than 1% of seeds nucleate in an hour (Supplementary Fig. 11E). We defined 125 nM as the threshold for the transition from the seeded nucleation and growth to the growth only regime.

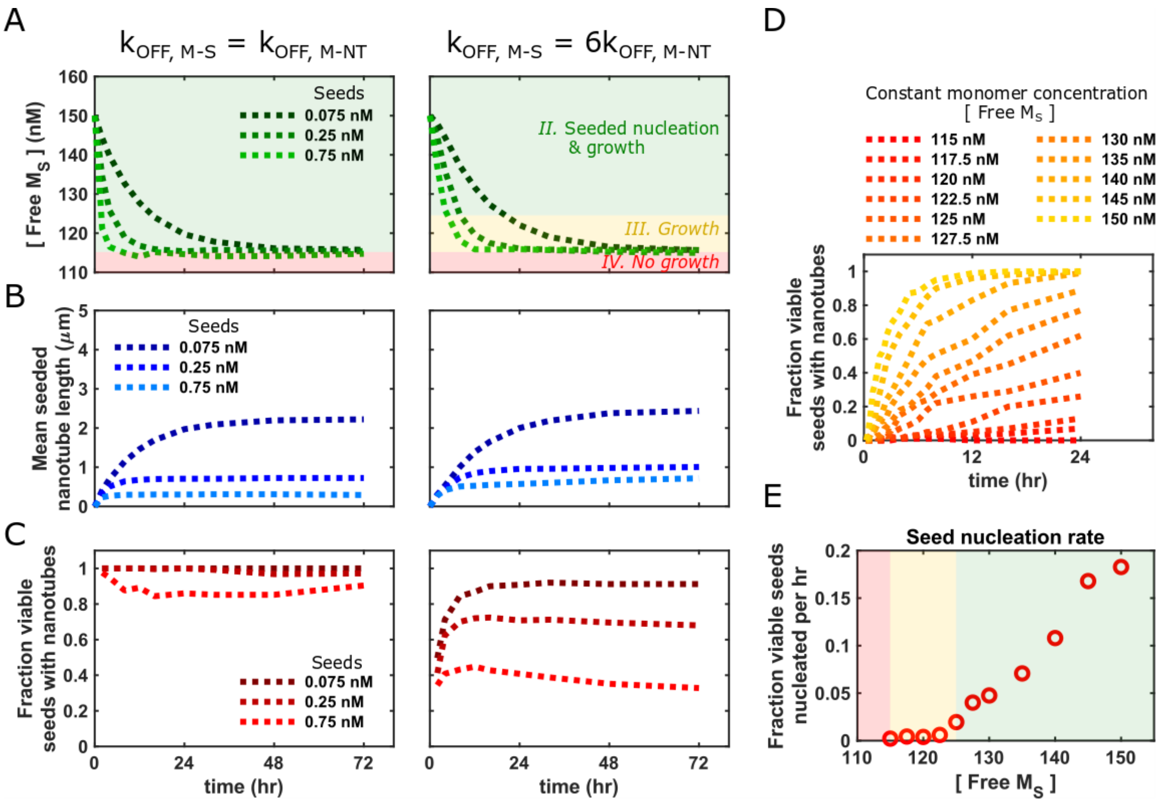


**Supplementary Figure 11:** Estimation of the monomer concentration where growth transitions from a seeded nucleation and growth regime to a growth only regime. (**A**-**C**) Simulation results for unregulated nanotube growth using 150 nM monomers without (left) and with (right) a higher off rate for monomers attached to seeds than monomers attached to nanotubes. These simulations indicate that the presence of the growth only regime (where additional seed nucleation is unlikely compared to nanotube growth) explains the dependence of seed nucleation yield on seed concentration (C). Simulations were otherwise conducted as described in Supplementary Note 4. (**D**) Fractions of viable seeds with nanotubes during growth with different initial monomer concentrations. The monomer concentrations were kept constant throughout the simulations (Supplementary Note 5). (**E**) Initial seed nucleation rates as a function of initial monomer concentrations. The seed nucleation rates were obtained by fitting the initial slopes (over the first 2 hours) of the simulation results in (D). Shaded regions in (A) and (E) correspond to the shaded regions in Figure 2C of the main text.

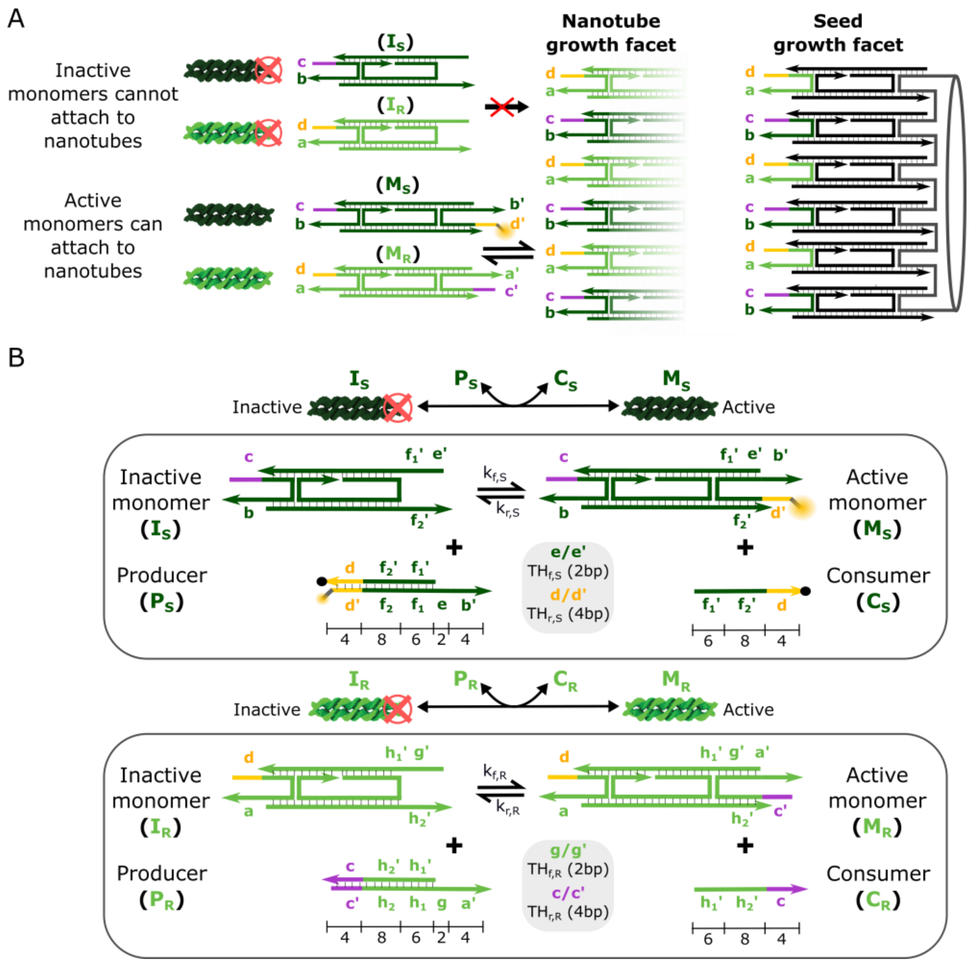


**Supplementary Figure 12:** The species used in reactions that buffer the concentrations of the S and R monomers. (**A**) Inactive monomers were designed by removing one of the strands that presents sticky ends from the active monomers. As a result, inactive monomers have only two of the four binding sites for other monomers and are unable to attach to the facet of the seeds used in this study or to a nanotube growing from these seeds. These inactive monomers then serve as a source for the production of active monomers. **(B**) Schematics of the monomer buffering reaction networks. Letters denote sequence domains with apostrophes indicating complementarity. Numbers indicated domain lengths. The toehold domains for the forward and reverse strand displacement reactions are indicated in the gray boxes.

**Supplementary Note 8: Calculating the equilibrium concentration of active monomers for a set of initial concentrations of buffering species**

The monomer buffering reaction network for one type of monomer is shown below:

$$I_i + P_i \overset{k_{f,i}}{\rightleftharpoons}_{k_{r,i}} M_i + C_i$$

Where *i* refers to either the R or S species, for a total of two buffering reaction networks. Assuming the two reaction rate constants for each buffering reaction and initial species concentrations are the same, the equilibrium concentrations of the species will be the same for the two reaction networks.

At equilibrium the forward and reverse reaction rates are equal. That is:

$$(3)\quad k_{f,i}[I_i]_{eq}[P_i]_{eq} = k_{r,i}[M_i]_{eq}[C_i]_{eq}$$

$$(4)\quad \frac{k_{f,i}}{k_{r,i}} = K_{eq,i} = \frac{[M_i]_{eq}[C_i]_{eq}}{[I_i]_{eq}[P_i]_{eq}}$$

$$(5)\quad [M_i]_{eq} = K_{eq,i}\frac{[I_i]_{eq}[P_i]_{eq}}{[C_i]_{eq}}$$

Where the *eq* subscript denotes equilibrium concentrations and $K_{eq,i}$ the equilibrium constant. For a given set of initial concentration of the monomer buffering species denoted using a o subscript, the concentrations of each species at equilibrium can be defined with respect to the difference between the equilibrium concentration that species and its initial concentration. This difference will be denoted Δx. For this analysis we will assume the reaction will be driven forward to reach equilibrium but the same result would be obtained if we assumed the reaction proceeded in the opposite direction:

$$(6)\quad [M_i]_{eq} = [M_i]_o + \Delta x$$

$$(7)\quad [C_i]_{eq} = [C_i]_o + \Delta x$$

$$(8)\quad [I_i]_{eq} = [I_i]_o - \Delta x$$

$$(9)\quad [P_i]_{eq} = [P_i]_o - \Delta x$$

Substituting Supplementary Equations 6-9 into Supplementary Equation 5 yields:

$$(10)\quad [M_i]_o + \Delta x - K_{eq}\frac{([I_i]_o - \Delta x)([P_i]_o - \Delta x)}{[C_i]_o + \Delta x} = 0$$

For a given equilibrium constant and set of initial concentrations, Supplementary Equation 10 can be solved for Δx and Δx can be plugged into Eqs. 6-9 to get the final equilibrium concentrations of each species.

This method was used to calculate the setpoint of the monomer buffering reaction network for a given set of initial species concentrations. Unless otherwise stated, $K_{eq} = 0.01$ was used for these calculations based on the assumption that the forward reaction rate constant ($k_f$) will be two orders of magnitude lower than the reverse reaction rate constant ($k_r$) since the forward reaction proceeds *via* a 2-base toehold mediated strand displacement process and the reverse reaction proceeds *via* a 4-base toehold mediated strand displacement process[7].

**Supplementary Note 9: Stochastic kinetic simulations of buffer-regulated nanotube growth**

To model buffer-regulated nanotube growth, the monomer buffering reactions were added to the stochastic model presented in Supplementary Note 4. The set of reactions allowed within this stochastic kinetic model of buffer-regulated nanotube growth are shown in Supplementary Fig. 13. The forward and reverse buffering reaction rate constants were assumed to be the same for each buffering reaction network, and were set at $k_{f,R} = k_{f,S} = 1x10^2\ M^{-1}s^{-1}$ and $k_{r,R} = k_{r,S} = 1x10^4\ M^{-1}s^{-1}$ , respectively. These values match previously measured rates constants for toehold-mediated strand displacement reactions involving toeholds with the same lengths[7]. In the simulation whose results are presented in Figure 4 of the main text, for both R and S monomers, the initial inactive monomer and $P_i$ concentrations were each set at 5.5 µM and the initial $C_i$ concentrations were set at 1.69 µM. These values set the monomer concentration setpoints to each be 155 nM (Supplementary Note 8). The initial active monomer concentration was 0. The reaction rates for nanotube growth presented in Supplementary Note 4 were used, *i.e.* $k_{ON} = 2x10^5\ M^{-1}s^{-1}$, $k_{OFF,\ M\text{-}NT} = 0.0231\ s^{-1}$, and $k_{OFF,\ M\text{-}S} = 6^*\ k_{OFF,\ M\text{-}NT}$.

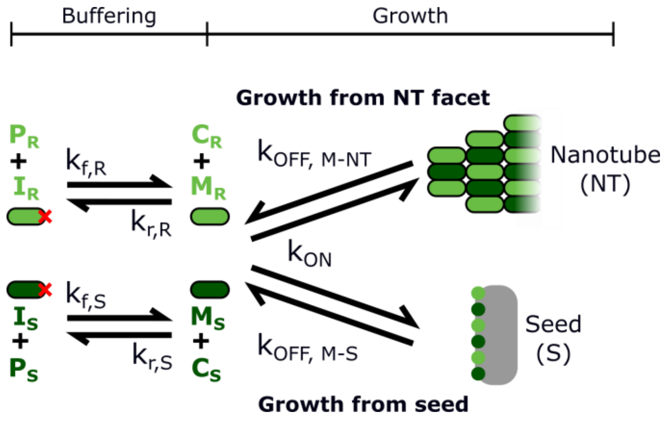


**Supplementary Figure 13:** Reactions used in the stochastic kinetic model of buffer-regulated growth. Active monomers can reversibly bind to sites on a growing nanotube or a seed face (right) where an active monomer can attach *via* two sticky ends as described in Supplementary Note 4. Buffering reactions between active monomers, and the respective inactive monomers ($I_i$), $P_i$, and $C_i$ species (left) are also modeled.

Simulations of buffer-regulated nanotube growth using the reaction rates and initial concentrations described above indicate that buffering should reduce the rate at which the active monomer concentration decreases during growth. That said, the monomer concentration will still decrease and, the higher the load, the faster this decrease in active monomer concentration will proceed (Supplementary Fig. 14A). The equilibrium active monomer concentrations (Supplementary Note 8) over the course of the simulations are virtually identical to the concentrations of the monomers over the course of the simulations, indicating that the monomer concentration remains at equilibrium with respect to the inactive monomers, $P_i$ and $C_i$ throughout the growth process (solid lines in Supplementary Fig. 14A). The concentrations of inactive monomers, $P_i$ and $C_i$ also remain at equilibrium with respect to the buffering reaction (Supplementary Fig. 14B). Thus, the decreases in the active monomer concentrations during growth are caused by the change in monomer concentration setpoint that occurs as the concentrations of the buffering species change during growth (Supplementary Fig. 14C). The rate of setpoint change is proportional to the rate at which monomers are consumed which is determined by the seed concentration (*i.e.* load) used for growth (Fig. 4D of the main text). These results indicate that buffer-regulated growth proceeds until the setpoint value drops to the critical concentration for growth.

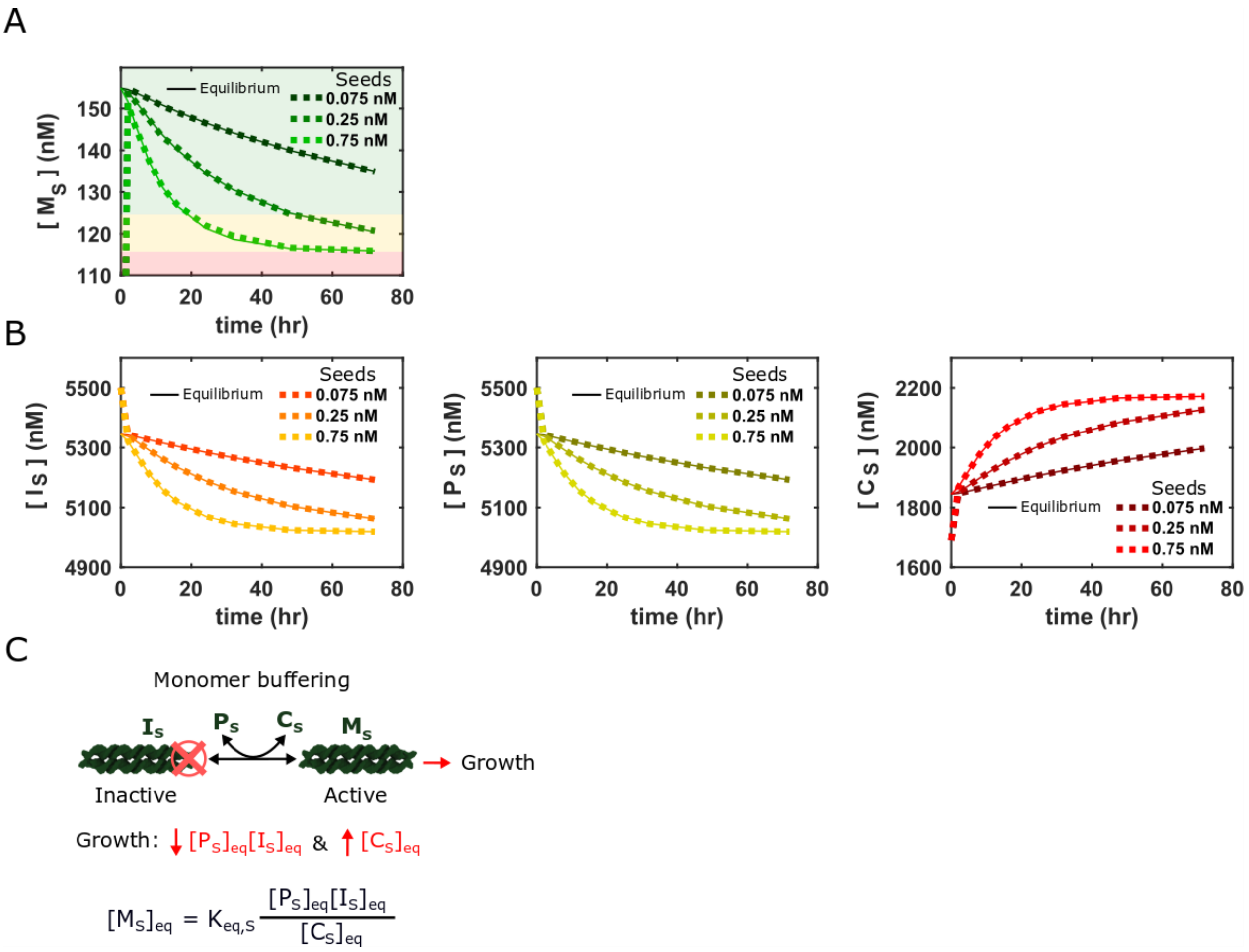


**Supplementary Figure 14:** The concentrations of active monomers and other buffering species change during simulations of regulated nanotube growth. (**A**) The concentrations of active S monomer during simulations of regulated growth. (**B**) Inactive monomer ($I_S$), $P_S$, and $C_S$ concentrations during simulations of regulated growth. The R monomer buffering species follow analogous trajectories. Dashed lines represent simulation results and solid lines represent the concentrations of species expected if the monomer buffering reaction network were at equilibrium given the concentrations of the monomer buffering species at each time point in the simulations (Supplementary Note 8). Shaded regions in (A) represent the different growth regimes from Figure 2C of the main text. Simulations were conducted as described in Supplementary Note 9. (**C**) Schematic depicting how changes in the concentrations of the buffering species during growth result in a decrease in the active monomer setpoint concentration.

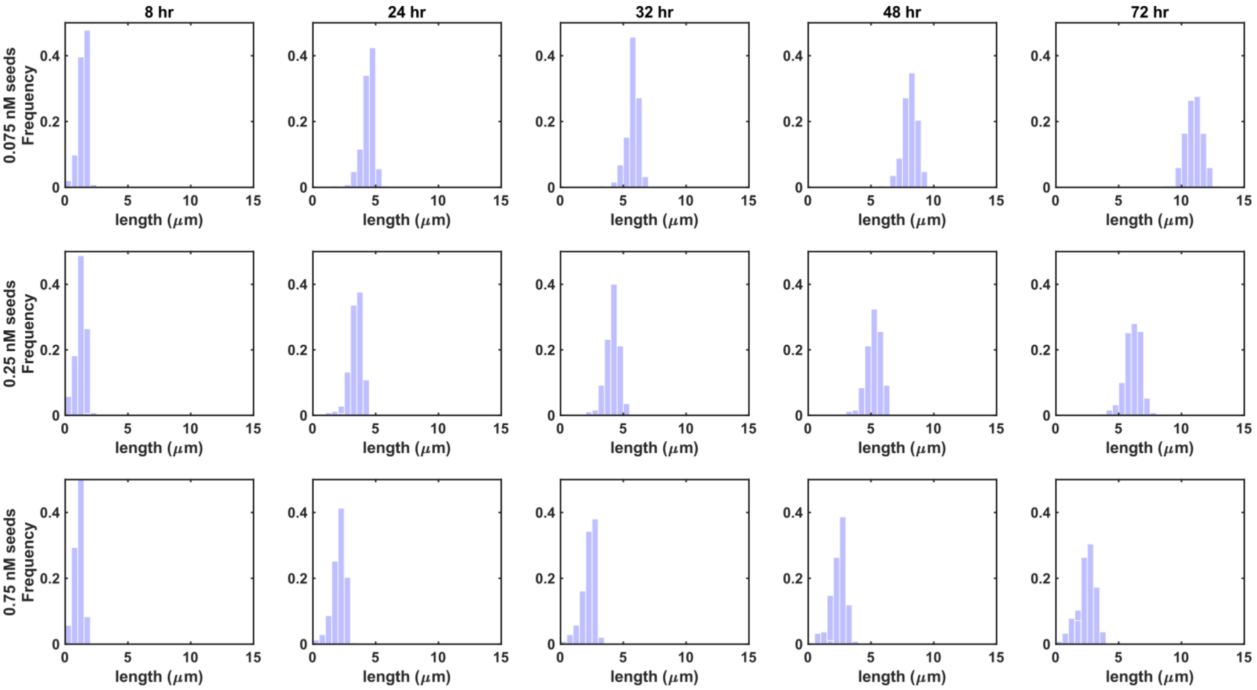


**Supplementary Figure 15:** Nanotube length distributions for buffer-regulated growth predicted by simulations. Simulations were conducted as described in Supplementary Note 9. Coefficient of variation values for the length distributions (dispersity) are in Supplementary Table 7.

**Supplementary Table 7:** Dispersity of nanotube length distributions computed as the coefficient of variation (CV) from the simulation results in Supplementary Figure 15.

| | **0.075 nM seeds** | **0.25 nM seeds** | **0.75 nM seeds** |
|---|---|---|---|
| **8 hr** | 25% | 31% | 30% |
| **24 hr** | 11% | 16% | 25% |
| **32 hr** | 9% | 13% | 24% |
| **48 hr** | 7% | 12% | 25% |
| **72 hr** | 6% | 11% | 28% |

A

| $[I_i]_o=[P_i]_o$=5.5 µM | |
|---|---|
| $[C_i]_o$ | Theor. $[M_i]_{eq}$ |
| 1.00 µM $C_i$ | 227 nM $M_i$ |
| 1.25 µM $C_i$ | 195 nM $M_i$ |
| 1.50 µM $C_i$ | 170 nM $M_i$ |
| 1.69 µM $C_i$ | 155 nM $M_i$ |

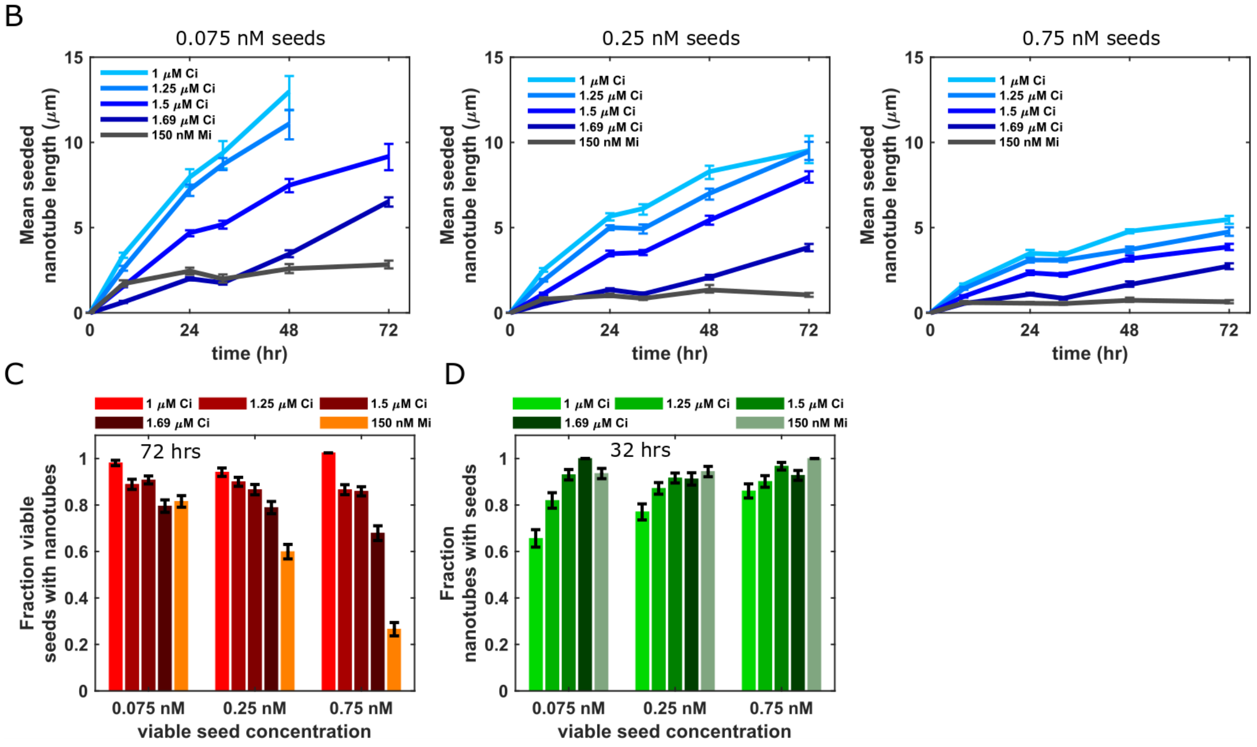


**Supplementary Figure 16:** Regulated nanotube growth with different $C_i$ concentrations. Reactions were conducted with $[I_i]_o$ = $[P_i]_o$ = 5.5 µM and different initial $C_i$ concentrations. (**A**) Table of the initial $C_i$ concentrations and the predicted active monomer setpoint concentrations for the initial buffering species concentrations calculated as described in Supplementary Note 8. (**B**-**D**) Measured mean seeded nanotube lengths (B), fractions of viable seeds with nanotubes (C), and fractions of nanotubes with seeds (D) for regulated nanotube growth for the concentrations of monomer buffering species given in (A). The sample sizes for every timepoint of each sample are tabulated in Supplementary Note 14. The mean lengths of nanotubes after 72 hours for the 0.075 nM seeds samples with $C_i$ concentrations of 1 µM and 1.25 µM are not shown as longer nanotubes broke during imaging, which prevented accurate determination of average nanotube lengths (Supplementary Fig. 18). $C_i$ concentrations of 1 µM and 1.25 µM produce nanotubes of similar lengths (B) and similar seed nucleation yields (C) across all seed concentrations, but the process with 1.25 µM $C_i$ produced much less unseeded nanotube growth with 1 µM $C_i$ (D). Error bars represent 95% confidence intervals.

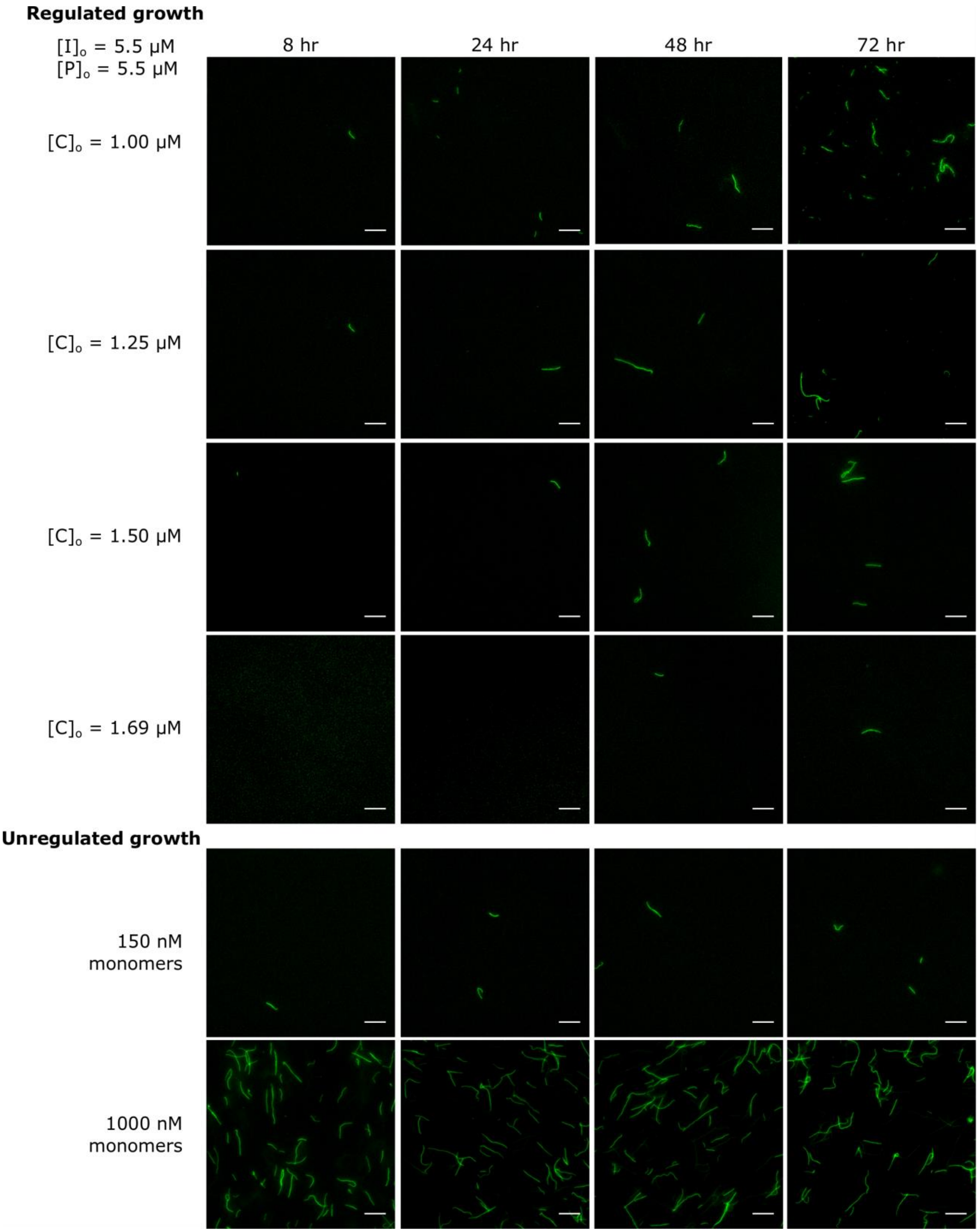


**Supplementary Figure 17:** Fluorescence micrographs of nanotube growth in the absence of seeds. For all regulated growth experiments other than those with 1 μM Consumer, little nanotube growth without seeds was observed. Other than the unregulated 1000 nM monomers sample, which was diluted 200x, all images were taken after a 100x dilution of the sample (the same dilution used to image the 0.075 nM viable seed samples in Fig. 5 in the main text). Scale bars: 10 μm.

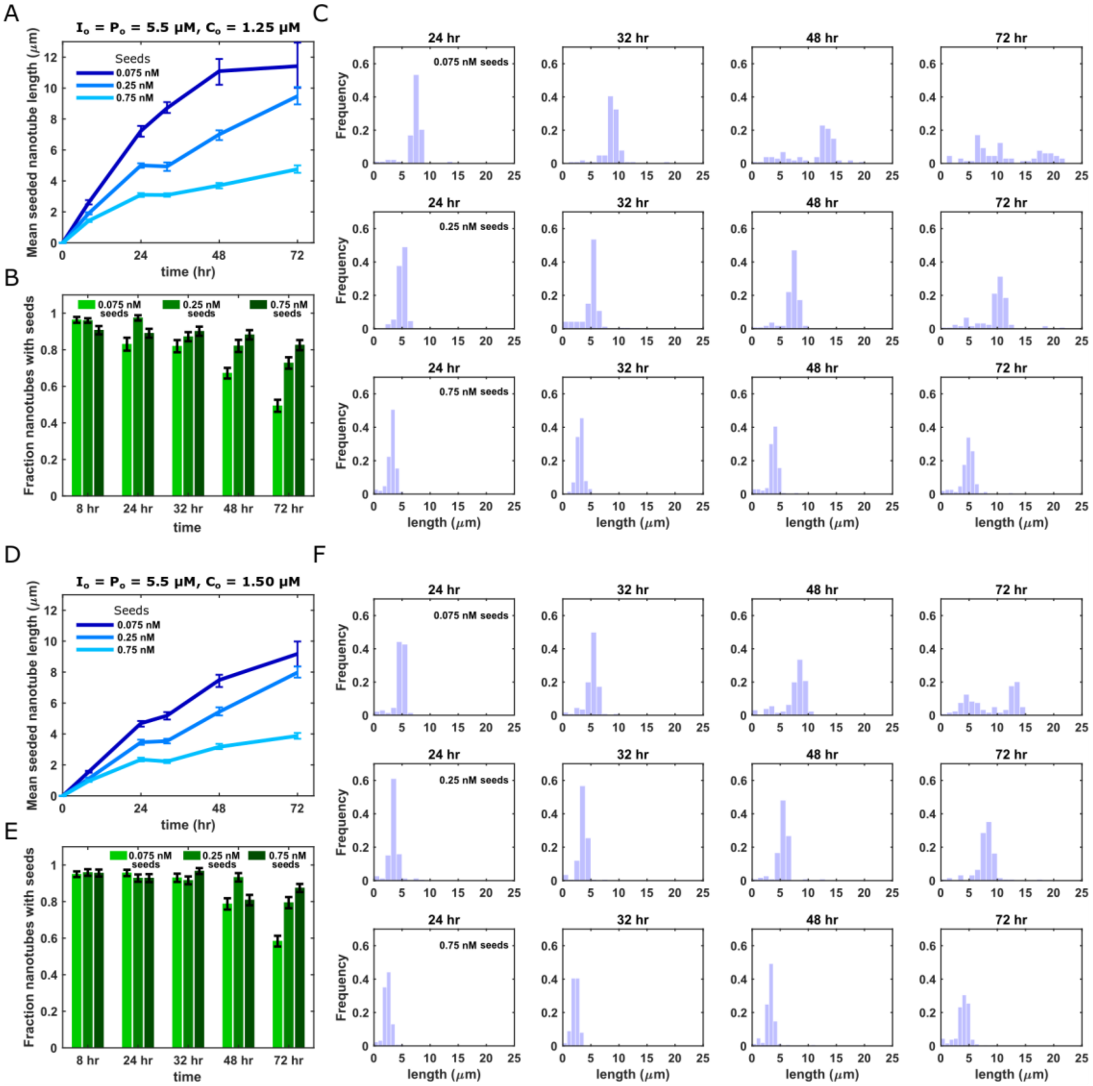


**Supplementary Figure 18:** Mean nanotube length quantification is skewed for samples with long nanotubes as longer nanotubes are prone to breaking during imaging. (**A**-**C**) Quantification of mean seeded nanotube lengths **(A)**, fraction of nanotubes with seeds **(B)**, and nanotube length distributions **(C)** during regulated nanotube growth with $[I_i]_o = [P_i]_o = 5.5$ μM and $[C_i]_o = 1.25$ μM for both monomer types as measured using the protocol in the Methods of main text where aliquots of samples were added to glass coverslips for imaging. In the sample containing 0.075 nM viable seeds, after 48 hrs when the nanotubes are roughly 10 μm long, there is a significant drop in the fraction of nanotubes with seeds (B) and shorter nanotubes start to arise in the length distributions (C). Hardly any growth is observed with these monomer buffering conditions in the absence of seeds (Supplementary Fig. 17) so the hypothesis that these trends result from homogeneous nucleation of nanotubes and/or nanotube ripening as these new tubes emerge is not consistent with these measurements. Instead, these trends seem to be a function of the nanotubes achieving sufficient length: these trends are not observed at time points or at seed concentrations when nanotubes are shorter. Long nanotubes would be more prone to breakage during pipetting or during adsorption to slides: we did observe that the long nanotubes in these samples were prone to breaking as they attach to the coverslip surface during imaging. Because nanotube breaking would skew measurements of nanotube length and the fractions of nanotubes with seeds, measurements from these time points were omitted from further analysis. Looking at the length distributions of the sample with 0.075 nM viable seeds after 72 hrs (C, top panel), there are two distributions of seeded nanotube lengths, one centered around 18 μm and another between 5 – 10 μm (where nanotubes are short enough to that they do not break), suggesting that in the absence of nanotubes breaking, the actual mean seeded nanotube length after 72 hrs for 0.075 nM viable seeds is around 18 – 20 μm. (**D-F)** Decreases in fractions of nanotubes with seeds and the emergence of seeded nanotubes also occur as nanotubes grow long with $[I_i]_o = [P_i]_o = 5.5$ μM and $[C_i]_o = 1.5$ μM for both monomer types. Error bars represent 95% confidence intervals.

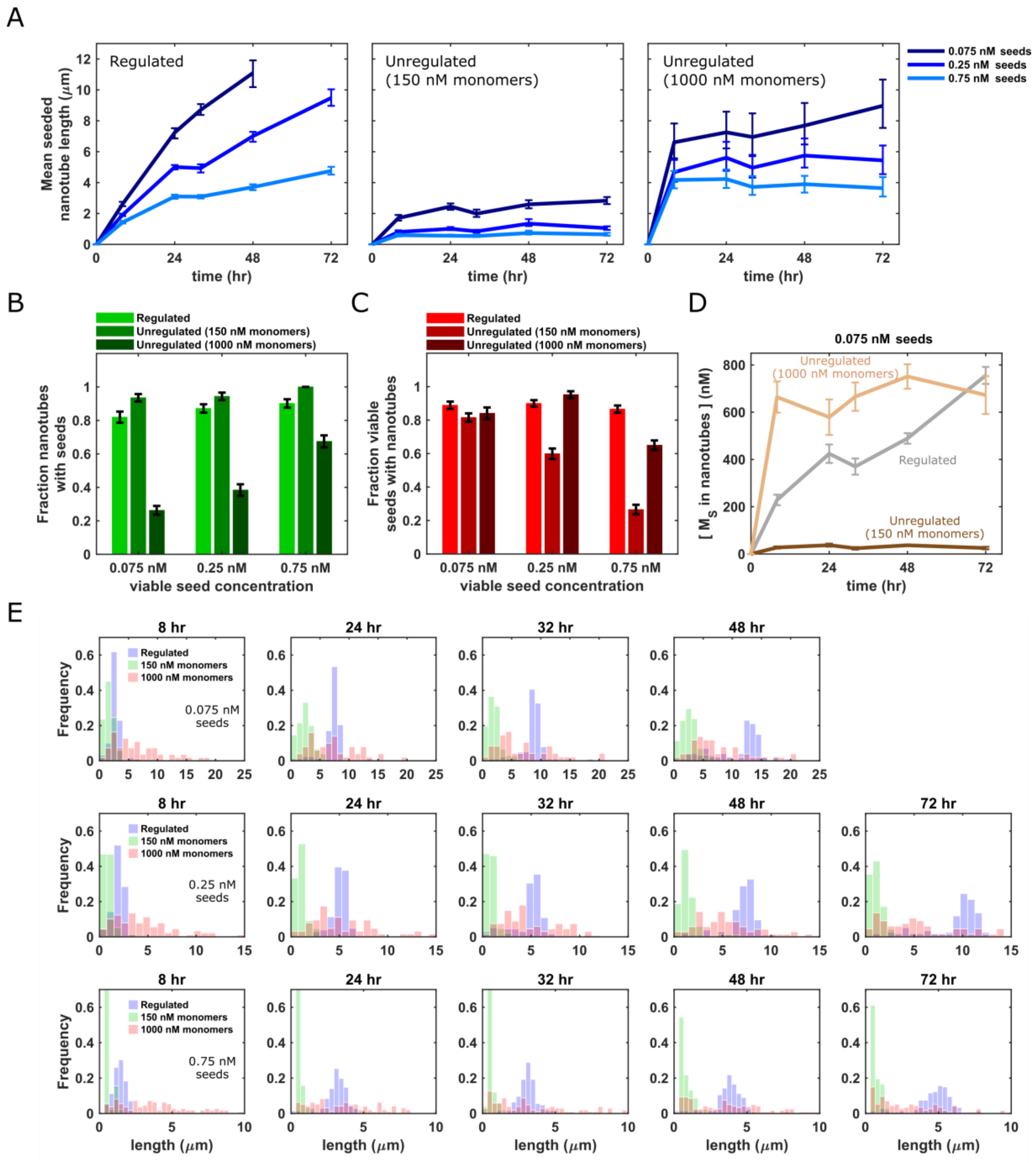


**Supplementary Figure 19:** Comparisons of the kinetics of regulated growth to the kinetics of unregulated growth with 150 nM or 1000 nM monomers. (**A**) Mean seeded nanotube lengths during regulated or unregulated growth at different seed and monomer concentrations. Error bars represent 95% confidence intervals from bootstrapping. (**B**,**C**) Fractions of nanotubes with seeds (B) and active seeds with nanotubes (C). Error bars represent 95% confidence intervals of proportions. (**D**) Concentration of S monomers incorporated in nanotubes during buffer-regulated and unregulated growth. Error bars represent standard deviation across images. The 1000 nM monomer sample may have lower S monomer incorporation than expected (roughly 900 nM S monomers should incorporate) because the measurement of concentration is a measure of nanotube lengths that assumes that each nanotube is 6 monomers in circumference (Supplementary Note 14). Unseeded nanotubes formed at high monomer concentrations often have larger circumferences, which would undercount incorporation[1,8]. (**E**) Histograms of seeded nanotube lengths during growth. Coefficient of variation values for the histograms are in Supplementary Table 8. The sample sizes for every timepoint of each sample are tabulated in Supplementary Note 14. Regulated growth experiments conducted with $[I_i]_o = [P_i]_o = 5.5$ μM and $[C_i]_o = 1.25$ μM for both monomer types.

**Supplementary Table 8:** Dispersity of nanotube length distributions computed from the coefficient of variation (CV). CV values computed from the experimental results in Supplementary Figure 19 and are presented as percentages. Buffer: buffer-regulated growth, UR 150: unregulated growth with 150 nM monomers, UR 1000: unregulated growth with 1000 nM monomers. The 24 hour timepoint for 0.75 nM seeds was presented in the main text.

| | 0.075 nM seeds | | | 0.25 nM seeds | | | 0.75 nM seeds | | |
|---|---|---|---|---|---|---|---|---|---|
| | **Buffer** | **UR 150** | **UR 1000** | **Buffer** | **UR 150** | **UR 1000** | **Buffer** | **UR 150** | **UR 1000** |
| **8 hr** | 30% | 54% | 67% | 29% | 54% | 75% | 35% | 50% | 72% |
| **24 hr** | 23% | 49% | 57% | 15% | 53% | 66% | 25% | 40% | 71% |
| **32 hr** | 21% | 62% | 69% | 34% | 53% | 60% | 20% | 47% | 86% |
| **48 hr** | 40% | 50% | 63% | 23% | 73% | 65% | 29% | 55% | 78% |
| **72 hr** | NA | 48% | 57% | 33% | 53% | 72% | 31% | 44% | 93% |

## Supplementary Note 10: Simulations of nanotube growth at a higher setpoint corresponding to $C_i$ = 1.25 µM

In the simulations presented in Figure 4 of the main text, we simulated buffer-regulated growth with $[I_i]_o$ = $[P_i]_o$ = 5.5 µM and $[C_i]_o$ =1.69 µM. However, experimentally we used a lower $[C_i]_o$ (1.25 µM). This lower value of $[C_i]_o$ could have resulted in a higher initial monomer setpoint concentration (predicted to be 195 nM), which could explain why we found more monomers were incorporated into nanotubes in experiments (Fig. 5E of the main text) after buffer-regulated nanotube growth than were in simulations where $[C_i]_o$ =1.69 µM. To determine the difference in the extent of growth that should be expected in experiments, we conducted simulations of buffer-regulated growth with $[C_i]_o$ =1.25 µM (corresponding to an initial setpoint monomer concentration of 195 nM) and compared those simulation results to simulations of unregulated growth with 195 nM monomers (Supplementary Fig. 20).

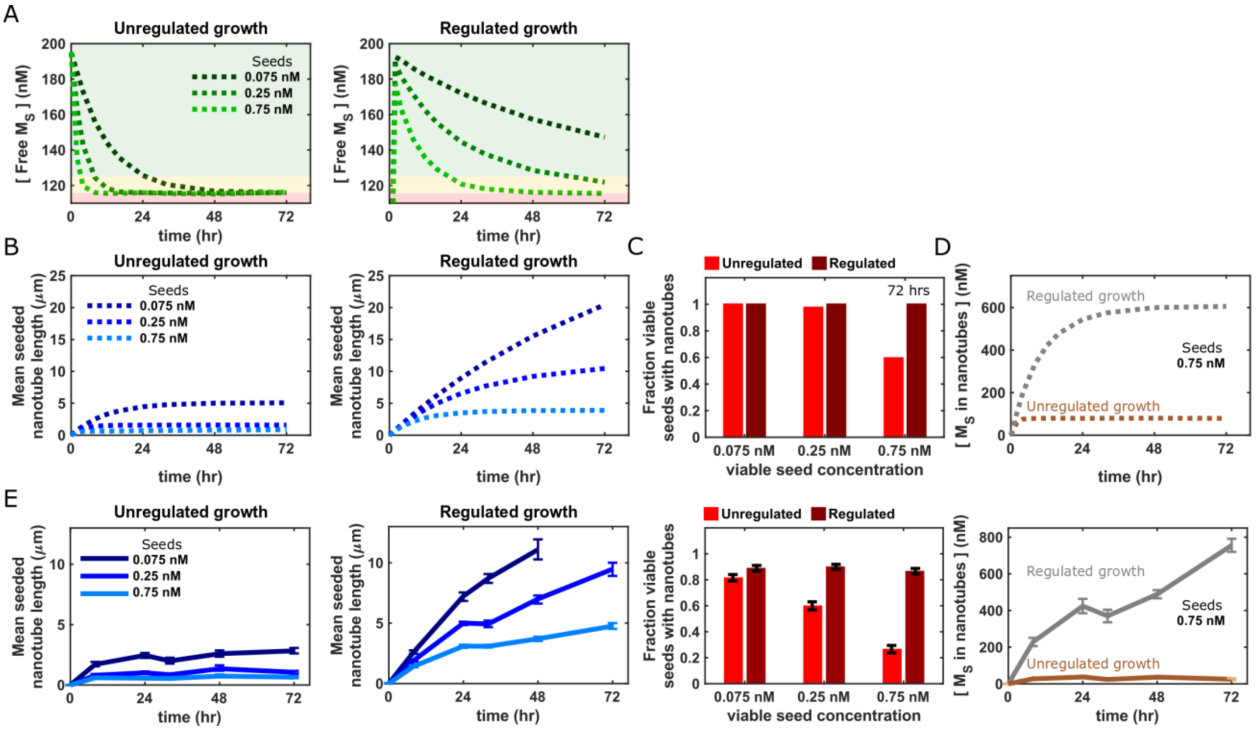


**Supplementary Figure 20:** Results of unregulated and regulated nanotube growth simulations at an initial monomer concentration of 195 nM. In unregulated growth simulations, the initial monomer concentrations were each 195 nM. Regulated growth simulations were conducted with $[I_i]_o$ = $[P_i]_o$ = 5.5 µM and $[C_i]_o$ =1.25 µM for both R and S monomers, resulting in a setpoint active monomer concentration of 195 nM. See Supplementary Note 9 for additional simulation details. (**A**) Free S monomer concentrations during growth. Shaded regions correspond to the growth regimes in Figure 2C. Only the concentration of the S monomer is shown; depletion of the two monomer types should happen at the same rate. (**B**) Mean lengths of seeded nanotubes during simulations of unregulated and regulated growth. (**C**) Fractions of viable seeds with nanotubes after 72 hours of growth predicted by simulations. (**D**) $[M_S]$ incorporated into nanotubes during growth. Buffer-regulated growth is predicted to incorporate >600 nM of monomers into nanotubes with $[C_i]_o$ =1.25 µM, much closer to the 750 nM that were incorporated in experiments than the $[M_S]$ predicted by the initial simulations in Figure 4D of the main text (360 nM after 72 hours) which used $[C_i]_o$ =1.69 µM. Further, the higher initial concentration of 195 nM in an unregulated growth process does not produce the same growth results observed experimentally for buffer-regulated growth. (**E**) For comparison, panels B, D, and E from Figure 5 of the main text are presented. Error bars for nanotube lengths represent 95% confidence intervals from bootstrapping. Error bars for fraction of seeds with nanotubes represent 95% confidence intervals of proportions. Error bars for [$M_S$ in nanotubes] represent standard deviation across images.

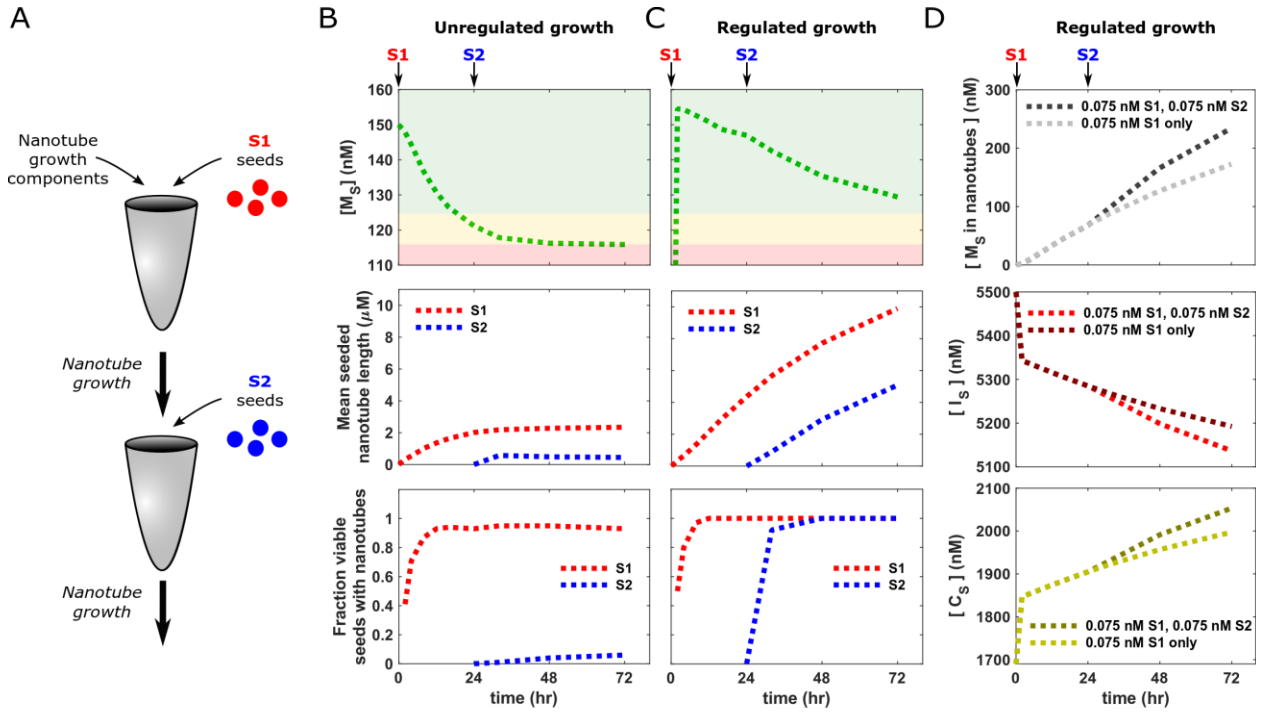


**Supplementary Figure 21:** Simulations of unregulated and regulated nanotube growth during a temporal increase in growth demand. (**A**) Schematic of the simulated experiment. The simulations are initiated with S1 seeds and nanotube growth species (active monomers for unregulated growth and buffering species for regulated growth) present. After 24 hours of simulated growth, S2 seeds are introduced into the simulations and growth can occur from either the S1 or S2 seeds. (**B**) Kinetics of nanotube growth and changes in monomer concentration predicted by simulations of unregulated growth initialized with 150 nM monomers. The simulations predict growth from the S1 seeds will cause the active monomer concentration to drop out of the seeded nucleation and growth regime (green shaded region) before S2 seeds are introduced at 24 hours and thus almost no growth from the S2 seeds is predicted. (**C**) Kinetics of nanotube growth and changes in monomer concentration predicted by simulations of buffer-regulated nanotube growth. The active monomer concentration is predicted to remain in the seeded nucleation and growth regime (green shaded region) throughout the growth process and thus nucleation and growth is predicted from both the S1 and S2 seeds. **(D)** The introduction of S2 seeds is predicted to increase the rate at which monomers are incorporated into nanotubes (top panel). To compensate for this increase in growth demand, the monomer buffering reaction network increases the rate of monomer production as evidenced by the increased rate of $I_S$ consumption and $C_S$ production (middle and bottom panels, respectively). Simulations were conducted using the growth parameters described in Supplementary Note 4 and the monomer buffering reaction network rate constants and species concentrations described in Supplementary Note 9.

**Supplementary Note 11: The reaction rates of monomer buffering may be much slower than the designed rates**

The stochastic kinetic model developed for buffer-regulated growth in Supplementary Note 9 predicted different rates of nanotube growth than those that were experimentally measured (Fig. 7A of the main text). Simulations predicted the same initial growth rates for all seed concentrations and a marked decrease in the growth rate after 24 hours for growth with 0.25 nM and 0.75 nM seeds. However, in experiments, we observed different initial (between 0-24 hours) growth rates for all the seed concentrations and the rate of nanotube growth remained fairly constant over the 72 hour growth period for all seed concentrations.

We adjusted different rate constants in simulations to try to elucidate the source of this discrepancy and to better understand the buffer-regulated growth process. One possibility was that rate of monomer attachment ($k_{ON}$) might be different for buffer-regulated compared to unregulated growth and that this difference caused the difference between simulation results and experiments. Such a difference might be caused by competition for active monomer attachment to growth sites by the buffering species. These buffering species would likely attach more transiently than active monomers but because these species are at very high concentrations, they could occupy a significant fraction of binding sites. To test this hypothesis, we simulated buffer-regulated growth using a few different $k_{ON}$ values. However, neither raising nor lowering the effective $k_{ON}$ improved the correspondence between the simulations and the experiments (Supplementary Fig. 22A-C).

We next considered the possibility that the monomer buffering reaction network rate constants ($k_f$ and $k_r$) used in the simulation might be higher than the true values for these rate constants. The rate constants we selected for the forward and reverse buffering reactions were designed to be large enough so that inactive monomers were replenished at rates higher than they could be depleted by the load imposed by the seeds at any of the concentrations we considered. Indeed, equilibrium analysis (Supplementary Note 8) of the active monomer concentration at each timepoint in our simulations confirmed monomer buffering to be faster than nanotube growth for our assumed monomer buffering reaction network rate constants (Supplementary Fig. 22D-F). However, if the rate constants for the monomer buffering reactions were much lower, we might expect to see slower nanotube growth rates as growth would deplete monomers faster than monomer buffering could replenish them. To test this theory, we lowered both of the monomer buffering reaction network rate constants by either one or two orders of magnitude ($k_f = 1x10^1$ $M^{-1}s^{-1}$ and $k_r = 1x10^3$ $M^{-1}s^{-1}$ or $k_f = 1x10^0$ $M^{-1}s^{-1}$ and $k_r = 1x10^2$ $M^{-1}s^{-1}$, respectively) and conducted our simulations again. The simulations performed with the rate constants that were two orders lower than we predicted correctly matched the different initial rates of growth seen in experiments. They further also recapitulated the maintenance of these rates of growth over the course of the growth experiments (Supplementary Fig. 23B). Conducting an equilibrium analysis on the simulated concentrations of the monomer buffering species ($I_i$, $P_i$, $M_i$, and $C_i$) during these simulations we found that, for $k_f = 1x10^0$ $M^{-1}s^{-1}$ and $k_r = 1x10^2$ $M^{-1}s^{-1}$, monomer buffering could not keep up with nanotube growth, so that the active monomer concentration was always lower than the setpoint concentration (Supplementary Fig. 23D). Additionally, the higher the seed concentration, the further the simulated active monomer concentrations are from their equilibrium values. These differences result in the different initial growth rates seen across the different seed concentrations.

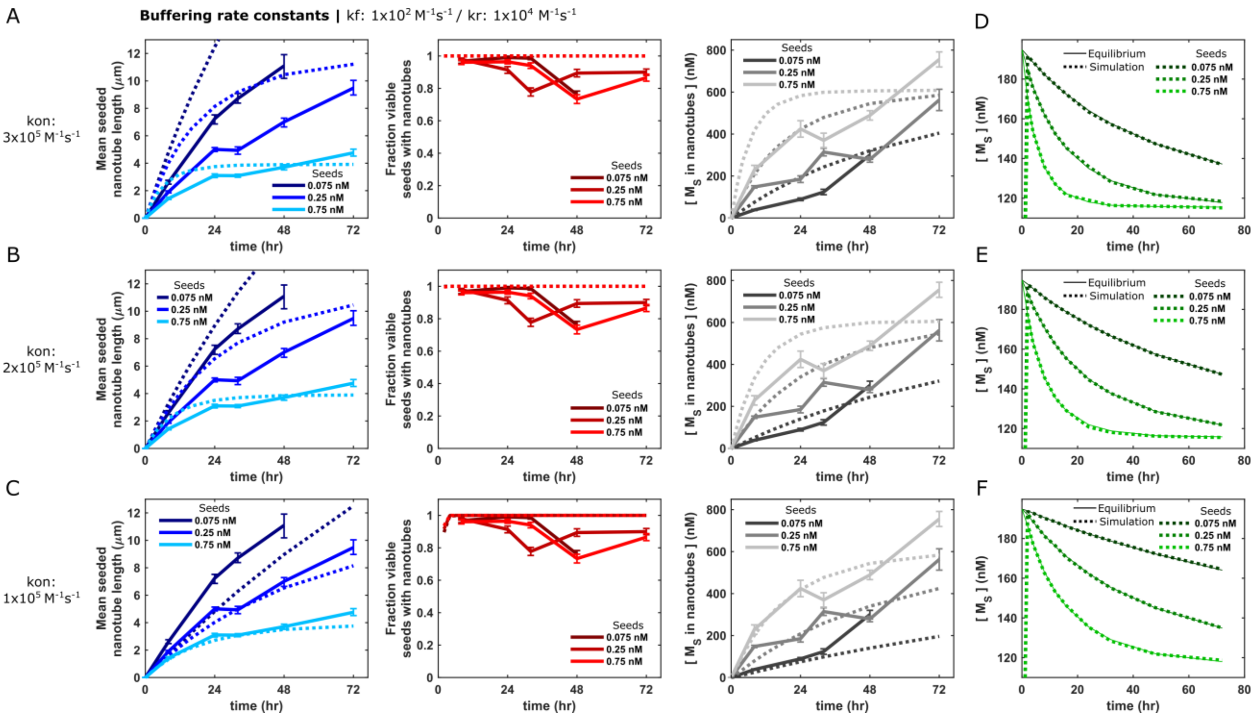


**Supplementary Figure 22:** (**A-C**) Comparison of the results of experimental (solid lines) measurements and kinetic simulation (dashed lines) of buffer-regulated nanotube growth with a $k_f$ of 1x10$^2$ M$^{-1}$s$^{-1}$ and a $k_r$ of 1x10$^4$ M$^{-1}$s$^{-1}$ for the monomer buffering reaction network rate constants. Simulations in (A), (B), and (C) were conducted with different values of $k_{ON}$. Experimental data is reproduced here from Figure 5 of the main text for comparison to simulation results. Error bars for nanotube lengths represent 95% confidence intervals from bootstrapping. Error bars for fraction of seeds with nanotubes represent 95% confidence intervals of proportions. Error bars for [$M_S$ in nanotubes] represent standard deviation across images. (**D-F**) S monomer concentrations during nanotube growth in the simulations in (A), (B), and (C), respectively. Only the S monomer concentrations are shown as the R monomer concentrations follow analogous trajectories. Solid lines represent the theoretical equilibrium values of the monomer buffering species during the course of the reaction (Supplementary Note 8). Stochastic simulations were otherwise conducted as described in Supplementary Note 9 with $[I_i]_o$ = $[P_i]_o$ = 5.5 μM and $[C_i]_o$ =1.25 μM for both monomer types.

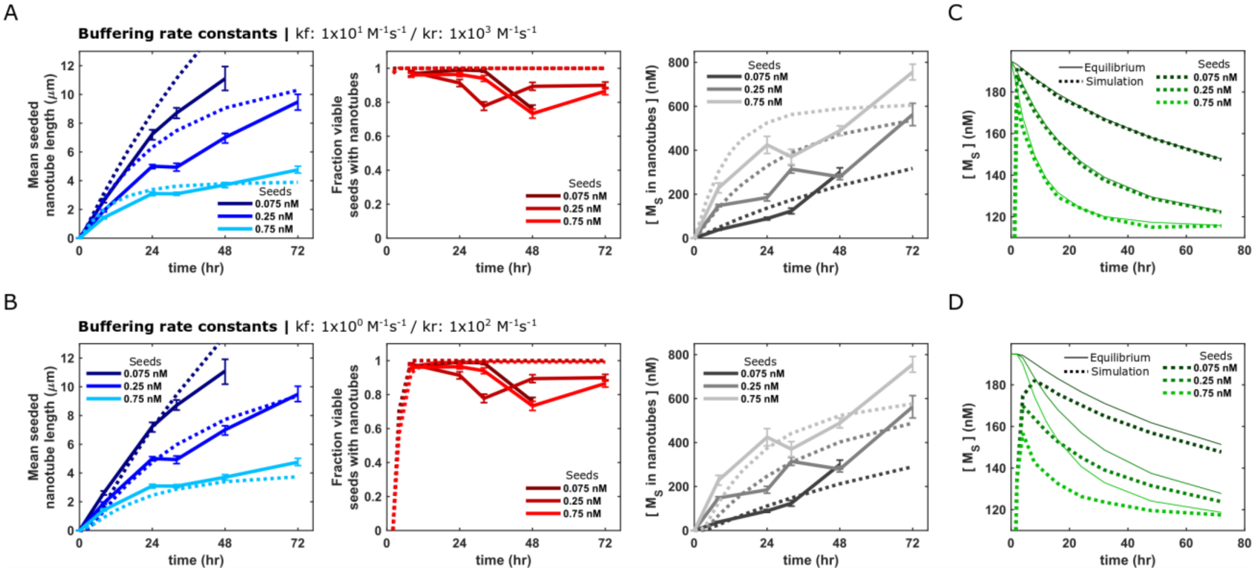


**Supplementary Figure 23:** (**A-B**) Comparison of the results of experimental (solid lines) measurements and kinetic simulation (dashed lines) of buffer-regulated nanotube growth with a $k_f$ of $1x10^1$ $M^{-1}s^{-1}$ and a $k_r$ of $1x10^3$ $M^{-1}s^{-1}$ (A) or $k_f$ of $1x10^0$ $M^{-1}s^{-1}$ and a $k_r$ of $1x10^2$ $M^{-1}s^{-1}$ (B) for the monomer buffering reaction network rate constants. Experimental data is reproduced here from Figure 5 of the main text for comparison to simulation results. Error bars for nanotube lengths represent 95% confidence intervals from bootstrapping. Error bars for fraction of seeds with nanotubes represent 95% confidence intervals of proportions. Error bars for [$M_S$ in nanotubes] represent standard deviation across images. (**C-D**) S monomer concentrations during nanotube growth in the simulations in (A) and (B), respectively. Only the S monomer concentrations are shown as the R monomer concentrations follow analogous trajectories. Solid lines represent the theoretical equilibrium values of the monomer buffering species during the course of the reaction (Supplementary Note 8). Stochastic simulations were otherwise conducted as described in Supplementary Note 9 with $[I_i]_o$ = $[P_i]_o$ = 5.5 μM and $[C_i]_o$ =1.25 μM for both monomer types.

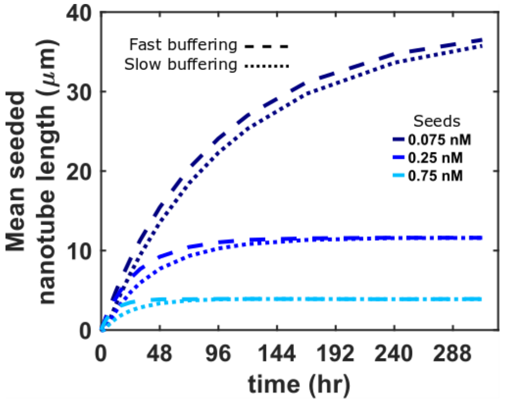


**Supplementary Figure 24:** Buffer-regulated growth with both fast and slow buffering reaction rate constants result in similar final mean nanotube lengths despite the fact that “fast” and “slow” buffering reaction rate constants differ by two orders of magnitude. Fast buffering simulations were conducted with $k_f$ of $1x10^2$ $M^{-1}s^{-1}$ and a $k_r$ of $1x10^4$ $M^{-1}s^{-1}$ and slow buffering simulations were conducted with $k_f$ of $1x10^0$ $M^{-1}s^{-1}$ and a $k_r$ of $1x10^2$ $M^{-1}s^{-1}$. Simulations were otherwise conducted as described in Supplementary Note 9 with $[I_i]_o$ = $[P_i]_o$ = 5.5 μM and $[C_i]_o$ =1.25 μM for both monomer types.

## Supplementary Note 12: Growth simulations with higher monomer buffering species concentrations

Supplementary Figure 25A,B show simulation results for regulated nanotube growth with initial inactive monomer and $P_i$ concentrations 1-, 2-, 5-, and 10-fold higher than the concentrations used in the simulations in Figure 4 of the main text. The $C_i$ concentrations were selected for each set of inactive monomer and $P_i$ concentrations so that the equilibrium concentration of active monomers would be 155 nM in each simulation. Supplementary Figure 25C shows how the depletion ratio changes as the initial concentrations of the monomer buffering species increase and Supplementary Figure 25D shows how the total concentration of active monomers that can be incorporated into nanotubes increases as the initial concentrations of the monomer buffering species increase.

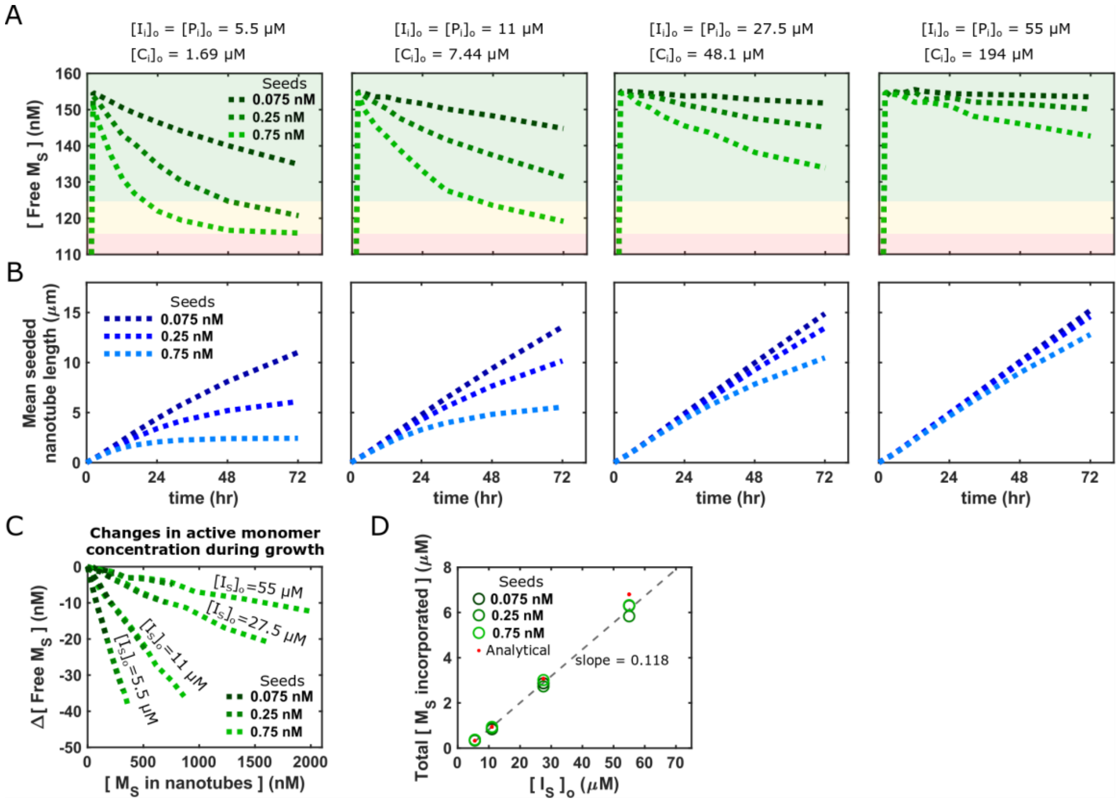


**Supplementary Figure 25:** Simulations of regulated nanotube growth with different initial concentrations of the monomer buffering species and a setpoint monomer concentration of 155 nM. (**A**,**B**) Concentrations of S monomers (A) and mean seeded nanotube lengths (B) during simulations of buffer-regulated nanotube growth. The initial concentrations of the monomer buffering species are as given above each pair of plots. Shaded regions indicate the different nanotube growth regimes (Fig. 2C of the main text). (**C**) Changes in free S monomer concentration observed in simulation as a function of total concentration of S monomers incorporated into nanotubes. The slopes of these lines represent the depletion ratio for different initial concentrations of the monomer buffering species. The depletion ratio is defined as the concentration of monomers incorporated into nanotubes divided by the decrease in the setpoint active monomer concentration. (**D**) The total concentration of S monomers that can be incorporated into nanotubes plotted for the different initial concentrations of inactive monomers. The $P_i$ and $C_i$ concentrations corresponding to each inactive monomer concentration are given in (A). The total concentration of monomers that can be incorporated into nanotubes before the buffer is exhausted is the difference between the initial setpoint concentration (155 nM) and the critical concentration (115 nM) divided by the depletion ratio for a given set of initial conditions. Thus the values plotted were obtained by dividing the 40 nM setpoint change by the slopes of the lines in (C). Red dots indicate the analytical values of the total amount of monomers that can be incorporated into nanotubes for a 40 nM setpoint change obtained from Equation 3 of the main text[9]. Simulations were conducted as described in Supplementary Note 9 except that the initial concentrations of the monomer buffering species were as presented in (A).

**Supplementary Note 13: Monomer buffering species affect nanotube growth**

Supplementary Figs. 26A and 27A, respectively, show that the presence of either $P_i$ complexes or inactive monomers at concentrations similar to those we used for the buffer-regulated growth experiments significantly decreased the amount of nanotube growth observed over 24 hours for an unregulated growth process. These results suggest that while buffering should regulate monomer concentration, the buffering species also introduce side reactions that can affect the growth process.

Supplementary Tables 9 and 10, respectively, estimate the percentage of growth sites that would be blocked for different $P_i$ concentrations and $P_i$ – growth site binding energies (Supplementary Table 9) and how much the active monomer concentration would decrease as a function of inactive monomer concentration and inactive-active monomer binding energies (Supplementary Table 10).

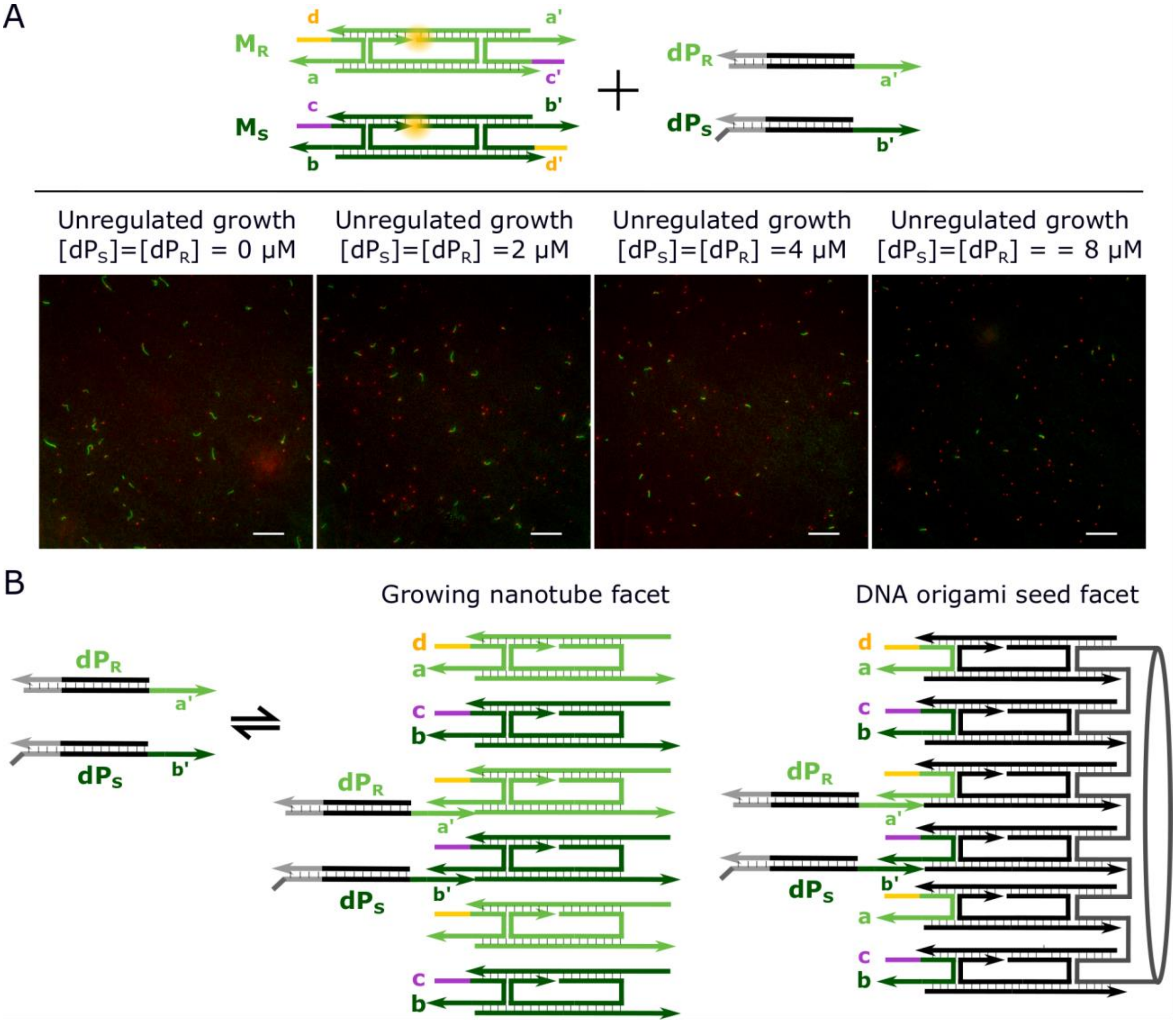


**Supplementary Figure 26:** $P_i$ complexes reduce the rate of nanotube growth by transiently blocking growth sites. (**A**) Fluorescence micrographs of nanotubes after unregulated nanotube growth with increasing concentrations of $P_i$. Nanotubes were grown with 10 pM seeds and images were taken after 24 hours of growth. Scale bars: 10 µm. In these experiments the $dP_i$ complexes were composed of strands with slightly different sequences than those used for $P_i$ (Supplementary Table 1). $dP_R$ consists of $dC_R$: `5' GTATGCATCTGTCCCTAG` and $dM_R$ – 2: `5' CTAGGGACAGATGCATACCGGCAT` and $dP_S$ consists of $dC_S$: `5' TTGATCCTTAAGCGGTTG` and $dM_S$ – 2: `5' TTCAACCGCTTAAGGATCAAAGAGGT`. The single-stranded sticky end regions of the dPi complexes were the same as the $P_i$ (Producer) complexes, so they could bind to nanotube facets and nanotube monomers, but the double-stranded portions of the $dP_i$ complexes were different than those for the Pi complexes such that the $dP_i$ complexes could not react with inactive monomers. Further, the $M_R$ – 3 and $M_S$ – 3 strands (from Supplementary Table 1) were modified with Cy3 on their 5' ends and an unmodified $M_S$ – 2 strand was used: `5' TCTGGTAGAGCACCACTGAGAGGT`, as in previous studies[1–3,10]. These modifications result in different thermodynamics of monomer binding than the monomers used in the rest of the study, where $M_S$ – 2 was modified with Cy3. As a result, nanotube growth occurred at monomer concentrations lower (50 nM monomers were used here) than in other experiments in this study. (**B**) Possible reversible interactions between the $dP_i$ complexes and the growth facet of a DNA nanotube or a seed. The reversible attachment of $dP_i$ complexes to the seeds or nanotube facets can reversibly block growth sites, slowing down the rate of nanotube growth.

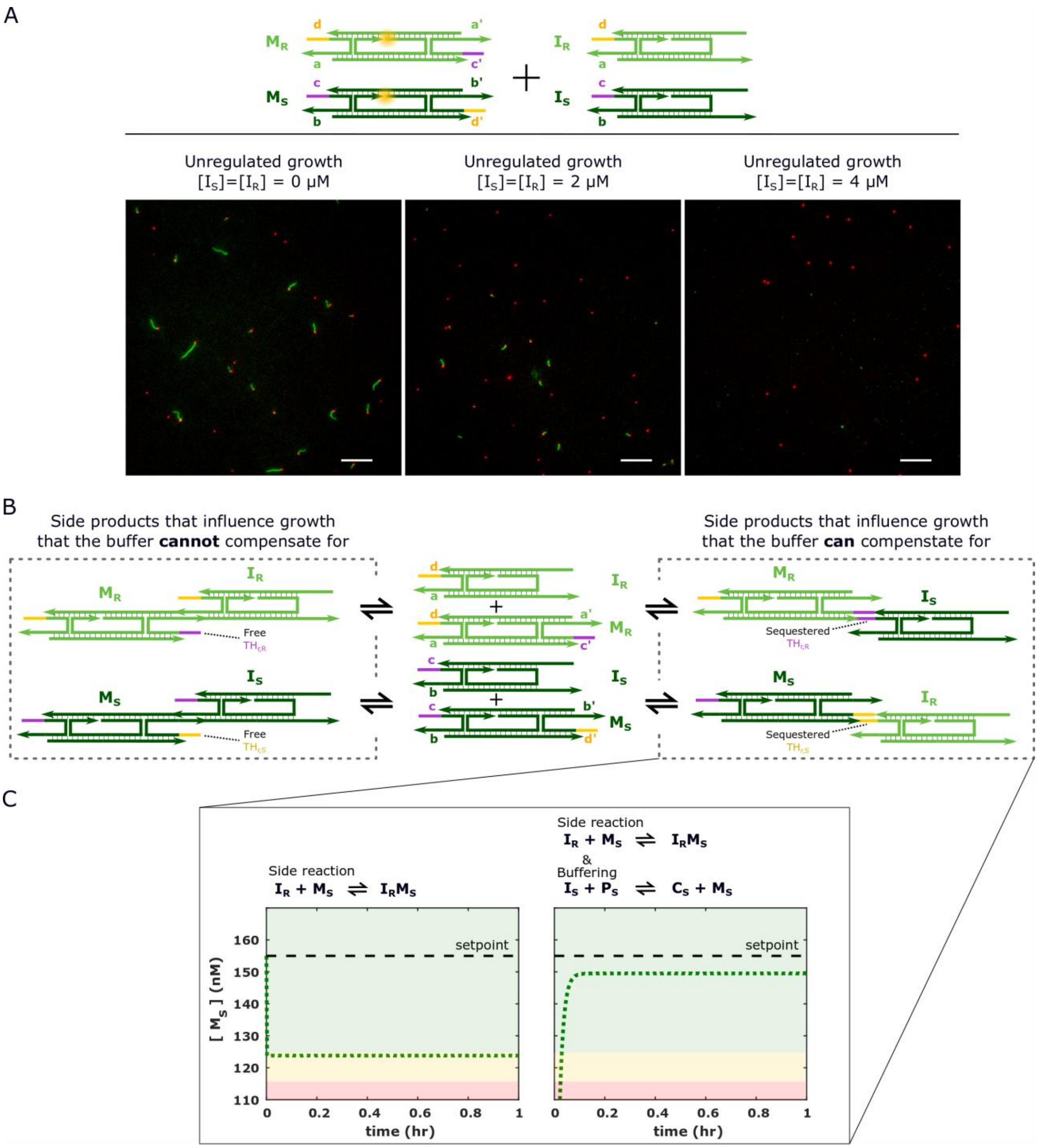


**Supplementary Figure 27:** Inactive monomers can reduce the rate of nanotube growth by transiently binding active monomers. (**A**) Fluorescence micrographs of nanotubes after unregulated growth in the presence of inactive monomers at the stated concentrations. Nanotubes were grown with 10 pM seeds and images were taken after 24 hours of growth. Scale bars: 10 µm. In these experiments the $M_R$ – 3 and $M_S$ – 3 strands (from Supplementary Table 1) were modified with Cy3 on their 5' ends and an unmodified $M_S$ – 2 strand was used: `5' TCTGGTAGAGCACCACTGAGAGGT`, as in previous studies[1–3,10]. As with the experimental results depicted in Supplementary Figure 26, these modifications changed the thermodynamics of monomer binding such that nanotube growth occurred at monomer concentrations lower (50 nM monomers were used here) than in other experiments in this study.

(**B**) Inactive monomers likely decrease the rate at which nanotubes grow because they can transiently bind to active monomers, which prevents active monomers from binding to growing nanotubes. These interactions thus effectively decrease the concentration of active monomers. The possible reversible interactions between inactive and active monomers are depicted. The reactions shown in the left box should not influence the rates of the monomer buffering reactions and therefore should not influence the monomer concentration setpoint. The presence of these reactions mean, however, that a fraction of the active monomers are unavailable for attachment to nanotubes because they are bound to inactive monomers. The reactions shown in the right box sequester the toehold used in the monomer buffering reactions (Supplementary Fig. 12). Monomers in this state cannot react with the $C_i$ species. Thus, the coupling of these reactions to the monomer buffering reaction reduces the net flux of the reverse buffering reaction. The buffer should act to counter this decreased flux by producing more active monomers. This counteraction could mitigate the effects of these side reactions on nanotube growth by restoring the monomer setpoint to a desired level as depicted in C. (**C**) Simulations of changes in active S monomer concentration over time in the presence of the side reactions in (B, right) without (left) and with (right) monomer buffering. Simulations were conducted in the absence of any nanotube growth. $I_R$ and $P_S$ were 5.5 µM and $C_S$ was 1.69 µM in simulations. The free energy of binding between $I_R$ and $M_S$ was 6.25 kcal/mol. Monomer buffering is predicted to adjust the monomer concentration to compensate for the side reactions in the right box of (B).

**Supplementary Table 9:** Computed percentages of growth sites blocked by $P_i$ complexes for different $P_i$ concentrations and free energies of $P_i$ – nanotube (NT) binding. For seed concentrations that are much lower than the $P_i$ concentrations, as in our experiments, the percentage of blocked growth sites is independent of seed concentration. For a $P_i$ – growth site binding energy of 6.5 kcal/mol, nearly 50% of the growth sites are predicted to blocked with 11 µM $P_i$ so $P_i$ concentrations above 10 µM will likely significantly reduce the rate of nanotube growth.

| $\Delta G^o_{P\text{-}NT}$ | $[Pi]_o$ = 0 µM | $[Pi]_o$ = 5.5 µM | $[Pi]_o$ = 11 µM | $[Pi]_o$ = 27.5 µM | $[Pi]_o$ = 55 µM |
|---|---|---|---|---|---|
| 5.0 kcal/mol | 0.0% | 2.9% | 5.6% | 12.9% | 22.8% |
| 6.0 kcal/mol | 0.0% | 14.1% | 24.7% | 45.1% | 62.2% |
| 6.5 kcal/mol | 0.0% | 28.0% | 43.7% | 66.0% | 79.5% |
| 7.0 kcal/mol | 0.0% | 47.8% | 64.7% | 82.1% | 90.2% |

**Supplementary Table 10:** Computed equilibrium active monomer concentrations of 150 nM of $M_s$ monomers when they can reversibly bind with $I_S$ and $I_R$ species (via the reactions $M_S + I_S \leftrightarrow M_S I_S$ and $M_S + I_R \leftrightarrow M_S I_R$ depicted in Supplementary Figure 27B) for different initial concentrations of $I_S$ and $I_R$ and for different free energies of these inactive – active monomer binding reactions, i.e. the sticky end strengths. Colors correspond to the growth regimes that these concentrations would fall into (these regimes are shown in Figure 2C of the main text). Even at moderate sticky end strengths (6.0 – 6.25 kcal/mol) and inactive monomer concentrations around 4 µM, the effective active monomer concentration becomes low enough that growth is no longer in the seeded nucleation and growth regime.

| $\Delta G^o_{I\text{-}NT}$ | $[I_S]_o = [I_R]_o$ = 0 µM | $[I_S]_o = [I_R]_o$ = 2 µM | $[I_S]_o = [I_R]_o$ = 4 µM | $[I_S]_o = [I_R]_o$ = 5.5 µM | $[I_S]_o = [I_R]_o$ = 11 µM |
|---|---|---|---|---|---|
| 5.0 kcal/mol | $[M_S]$ = 150 nM | $[M_S]$ = 147 nM | $[M_S]$ = 144 nM | $[M_S]$ = 142 nM | $[M_S]$ = 134 nM |
| 6.0 kcal/mol | $[M_S]$ = 150 nM | $[M_S]$ = 134 nM | $[M_S]$ = 121 nM | $[M_S]$ = 113 nM | $[M_S]$ = 91 nM |
| 6.25 kcal/mol | $[M_S]$ = 150 nM | $[M_S]$ = 127 nM | $[M_S]$ = 110 nM | $[M_S]$ = 100 nM | $[M_S]$ = 75 nM |
| 6.5 kcal/mol | $[M_S]$ = 150 nM | $[M_S]$ = 117 nM | $[M_S]$ = 96 nM | $[M_S]$ = 85 nM | $[M_S]$ = 59 nM |

**Supplementary Note 14: Image analysis**

The lengths of nanotubes, whether nanotubes had attached seeds and *vice versa* were determined from fluorescence micrographs using custom image analysis scripts in MATLAB. Five to six images were typically processed and analyzed for a specific sample at a given timepoint typically corresponding to at least 100 seeds and 100 nanotubes per sample at a given timepoint. The exact number of nanotubes and seeds analyzed in each experiment are tabulated in Supplementary Tables 11-13. Below is the workflow for the image analysis process.

*Detecting nanotubes and seeds in fluorescence micrographs*

A fluorescence micrograph of DNA nanotubes and a corresponding fluorescence micrograph of DNA origami seeds were imported simultaneously for analysis. Canny edge detection[11] was used to detect the edges of objects in both the DNA nanotube image and the DNA origami seed image to produce binary images of the object edges. The edges of the detected objects both images were then filled in with pixels using the *bwmorph( )* function in MATLAB. No further processing was done to the DNA origami seed image which we refer to as qS below.

For the DNA nanotube image, morphological operations were then applied with the *bwmorph( )* function in MATLAB to skeletonize all the detected objects to be 1 pixel in width. This processed nanotube image (referred to as qFSwN below) was used, in conjunction with the processed seed image (qS), for the quantification of the fraction of seeds with nanotubes (Supplementary Fig. 28B).

*Quantification of the fraction of seeds with nanotubes*

To quantify the fraction of seeds with nanotubes, the locations of the endpoints of each nanotube in the qFSwN nanotube image were determined. A radius (typically 2 to 4 pixels) around each of these endpoint locations was searched in the processed qS image and if a seed was found in the search radius, this seed was counted as having a nanotube attached to it (Supplementary Fig. 28B). The fraction of seeds with nanotubes from a set of a images for a given sample at a specific timepoint was then calculated as the total number of seeds that had a nanotube attached to them in those images divided by the total number of seeds for those images. The total number of seeds in each image was measured by counting all the individual objects in the processed qS image. To correct for 25% of the seeds being unviable for nucleation (Supplementary Note 6), the raw fraction of seeds with nanotubes was divided by 0.75 to yield the fraction of viable seeds with nanotubes that is presented in the figures. Error bars for the fraction of seeds with nanotubes represent the 95% confidence intervals of proportions ($CI = \pm 1.96\sqrt{p(1-p)/n}$). Supplementary Table 11 shows the number of viable seeds analyzed to quantify the fraction of viable seeds with nanotubes for every timepoint of each sample.

**Supplementary Table 11:** The number of viable seeds (sample size) analyzed to quantify the fraction of viable seeds with nanotubes for every timepoint of each experimental sample in this study. 25% more seeds were actually analyzed, however, the number of viable seeds represents the denominator in the calculation of fraction of viable seeds with nanotubes. See Supplementary Equation 13 for the formula to convert these seed numbers into concentrations.

| **Unregulated growth 150 nM monomers (Figures 2 and 5)** | | | | | |
|---|---|---|---|---|---|
| | 8 hr | 24 hr | 32 hr | 48 hr | 72 hr |
| 0.075 nM seeds | 125 | 167 | 147 | 137 | 182 |
| 0.25 nM seeds | 172 | 143 | 214 | 176 | 185 |
| 0.75 nM seeds | 152 | 228 | 227 | 164 | 177 |

| Unregulated growth 1000 nM monomers (Supplementary Figure 19) | | | | | |
|---|---|---|---|---|---|
| | 8 hr | 24 hr | 32 hr | 48 hr | 72 hr |
| 0.075 nM seeds | 95 | 85 | 91 | 70 | 83 |
| 0.25 nM seeds | 107 | 98 | 77 | 90 | 84 |
| 0.75 nM seeds | 204 | 170 | 169 | 219 | 211 |
| **Regulated growth with Ci = 1000 (Supplementary Figure 16)** | | | | | |
| | 8 hr | 24 hr | 32 hr | 48 hr | 72 hr |
| 0.075 nM seeds | 137 | 103 | 124 | 128 | 105 |
| 0.25 nM seeds | 134 | 188 | 116 | 129 | 128 |
| 0.75 nM seeds | 152 | 160 | 118 | 256 | 123 |
| **Regulated growth with Ci = 1250 (Figure 5)** | | | | | |
| | 8 hr | 24 hr | 32 hr | 48 hr | 72 hr |
| 0.075 nM seeds | 140 | 105 | 127 | 271 | 155 |
| 0.25 nM seeds | 251 | 136 | 207 | 132 | 182 |
| 0.75 nM seeds | 153 | 160 | 143 | 202 | 188 |
| **Regulated growth with Ci = 1500 (Supplementary Figure 16)** | | | | | |
| | 8 hr | 24 hr | 32 hr | 48 hr | 72 hr |
| 0.075 nM seeds | 209 | 155 | 146 | 160 | 212 |
| 0.25 nM seeds | 158 | 184 | 173 | 125 | 176 |
| 0.75 nM seeds | 119 | 176 | 144 | 191 | 234 |
| **Regulated growth with Ci = 1690 (Supplementary Figure 16)** | | | | | |
| | 8 hr | 24 hr | 32 hr | 48 hr | 72 hr |
| 0.075 nM seeds | 170 | 157 | 187 | 107 | 171 |
| 0.25 nM seeds | 140 | 198 | 152 | 200 | 180 |
| 0.75 nM seeds | 191 | 192 | 193 | 158 | 164 |
| **Figure 6a-c** | | | | | |
| | 6.5 hr | 24 hr | 32 hr | 48 hr | 72 hr |
| S1 regulated | 205 | 111 | 122 | 95 | 114 |
| S1 unregulated 150 nM | 188 | 293 | 75 | 185 | 203 |
| S2 regulated | NA | NA | 173 | 104 | 83 |
| S2 unregulated 150 nM | NA | NA | 56 | 81 | 25 |
| **Figure 6d-f** | | | | | |
| | 7 hr | 23 hr | 32 hr | 48 hr | 72 hr |
| No caps | 123 | 89 | 95 | 95 | 80 |
| 8hr caps | 114 | 106 | 87 | 95 | 38 |
| 24hr caps | 93 | 107 | 90 | 46 | 76 |

_Quantification of the fraction of nanotubes with seeds_

To quantify the fraction of nanotubes with seeds, the objects in the qFSwN nanotube image that extended past the boundary of the image were removed, since it could not be definitively determined whether nanotubes with one or both ends outside the image's field of view have attached seeds. We termed this processed nanotube image, in which nanotubes with one or both ends outside the image boundary are removed, qFNwS (Supplementary Fig. 28C). To calculate the fraction of the nanotubes with seeds, the locations of the endpoints of each nanotube in the qFNwS nanotube image were first determined. Then a radius (typically 2 to 4 pixels) around each of these endpoint locations was searched in the processed qS image. If a seed was found in the search radius of one of the ends of a nanotube, it was determined that this nanotube end had an attached seed (Supplementary Fig. 28C). The fraction of nanotubes in a set of images processed for a given sample at a specific timepoint was then calculated as the total number of nanotube ends that had a seed attached to them divided by the total number of nanotubes counted in the qFNwS nanotube image. Although samples were diluted such that most nanotubes were isolated in images, some nanotubes crossed over one another in samples with longer nanotubes (typically <25% of the counted nanotubes), resulting in branched objects with more than two endpoints in the processed images. Our method of counting the total number of nanotubes in an image took these crossings into account by calculating the total number of nanotubes as:

$$(11) \quad ceil\left(\frac{\#\ of\ endpoints}{2}\right)$$

Where ceil(x) rounds its argument (x) to the lowest integer greater than or equal to it. Using this formula, an object with two endpoints would be counted as a single nanotube, an object with three or four endpoints would be counted as two nanotubes, an object with five or six endpoints would be counted as three nanotubes, etc. Error bars for the fraction of nanotubes with seeds were reported as the 95% confidence intervals of proportions. Supplementary Table 12 shows the number of nanotubes analyzed to quantify the fraction of nanotubes with seeds for every timepoint of each sample.

**Supplementary Table 12:** The number of nanotubes (sample size) analyzed to quantify the fraction of nanotubes with seeds for every timepoint of each experimental sample in this study. The number of nanotubes here represent the denominator in the calculation of fraction of the nanotubes with seeds.

| **Unregulated growth 150 nM monomers (Figures 2 and 5)** | | | | | |
|---|---|---|---|---|---|
| | 8 hr | 24 hr | 32 hr | 48 hr | 72 hr |
| 0.075 nM seeds | 110 | 147 | 124 | 106 | 153 |
| 0.25 nM seeds | 115 | 119 | 107 | 100 | 113 |
| 0.75 nM seeds | 76 | 96 | 65 | 66 | 50 |
| **Unregulated growth 1000 nM monomers (Supplementary Figure 19)** | | | | | |
| | 8 hr | 24 hr | 32 hr | 48 hr | 72 hr |
| 0.075 nM seeds | 339 | 291 | 278 | 243 | 275 |
| 0.25 nM seeds | 229 | 180 | 198 | 177 | 173 |
| 0.75 nM seeds | 190 | 153 | 169 | 200 | 184 |
| **Regulated growth with Ci = 1000 (Supplementary Figure 16)** | | | | | |
| | 8 hr | 24 hr | 32 hr | 48 hr | 72 hr |
| 0.075 nM seeds | 158 | 118 | 160 | 160 | 171 |
| 0.25 nM seeds | 138 | 214 | 148 | 143 | 163 |
| 0.75 nM seeds | 151 | 153 | 129 | 251 | 142 |

| Regulated growth with Ci = 1250 (Figure 5) | | | | | |
|---|---|---|---|---|---|
| | 8 hr | 24 hr | 32 hr | 48 hr | 72 hr |
| 0.075 nM seeds | 137 | 112 | 133 | 262 | 231 |
| 0.25 nM seeds | 249 | 116 | 179 | 134 | 202 |
| 0.75 nM seeds | 161 | 164 | 142 | 161 | 189 |
| **Regulated growth with Ci = 1500 (Supplementary Figure 16)** | | | | | |
| | 8 hr | 24 hr | 32 hr | 48 hr | 72 hr |
| 0.075 nM seeds | 199 | 139 | 129 | 174 | 281 |
| 0.25 nM seeds | 122 | 168 | 167 | 119 | 175 |
| 0.75 nM seeds | 114 | 140 | 121 | 193 | 223 |
| **Regulated growth with Ci = 1690 (Supplementary Figure 16)** | | | | | |
| | 8 hr | 24 hr | 32 hr | 48 hr | 72 hr |
| 0.075 nM seeds | 144 | 121 | 133 | 80 | 135 |
| 0.25 nM seeds | 104 | 137 | 114 | 124 | 164 |
| 0.75 nM seeds | 143 | 151 | 152 | 134 | 115 |
| **Figure 6a-c** | | | | | |
| | 6.5 hr | 24 hr | 32 hr | 48 hr | 72 hr |
| Regulated | 198 | 108 | 272 | 207 | 229 |
| Unregulated 150 nM | 144 | 205 | 63 | 133 | 133 |
| **Figure 6d-f** | | | | | |
| | 7 hr | 23 hr | 32 hr | 48 hr | 72 hr |
| No caps | 145 | 99 | 113 | 133 | 100 |
| 8hr caps | 142 | 141 | 122 | 113 | 53 |
| 24hr caps | 111 | 100 | 111 | 54 | 84 |

*Quantification of nanotube lengths*

To quantify the lengths of the nanotubes, the qFNwS nanotube image was used since the lengths of nanotubes that extend beyond the boundary of the image could not be determined. For the purposes of measuring length, branched structures in the qFNwS nanotube images were removed since the lengths of nanotubes that cross over cannot be accurately determined. We termed this processed nanotube image qNL (nanotubes in Supplementary Fig. 28D). Since each nanotube in the qNL image was represented as a single pixel-width object, the length of each nanotube was calculated by measuring the number of pixels in the nanotube. In our images (Methods of the main text), each pixel is 170 nm by 170 nm. Each pixel horizontally or vertically connected to another pixel was thus considered as 170 nm of length. Each pixel diagonally connected to another pixel was considered as $\sqrt{2}*170$ nm. The mean length of the nanotubes for a specific sample at a given timepoint was calculated from all the nanotube lengths obtained across all the images processed for that sample and timepoint. For samples with seeds, only the lengths of nanotubes attached to seeds were measured. Error bars for nanotube lengths represent 95% confidence intervals computed using the *bootstrapci( )* function in MATLAB. Supplementary Table 13 shows the number of nanotubes analyzed to quantify nanotube length for every timepoint of each sample.

**Supplementary Table 13:** The number of nanotubes (sample size) analyzed to quantify the mean nanotube length for every timepoint of each experimental sample in this study.

| **Unregulated growth 150 nM monomers (Figures 2 and 5)** | | | | | |
|---|---|---|---|---|---|
| | 8 hr | 24 hr | 32 hr | 48 hr | 72 hr |
| 0.075 nM seeds | 102 | 130 | 107 | 88 | 135 |
| 0.25 nM seeds | 96 | 108 | 87 | 88 | 93 |
| 0.75 nM seeds | 45 | 63 | 50 | 46 | 36 |
| **Unregulated growth 1000 nM monomers (Supplementary Figure 19)** | | | | | |
| | 8 hr | 24 hr | 32 hr | 48 hr | 72 hr |
| 0.075 nM seeds | 55 | 50 | 48 | 51 | 41 |
| 0.25 nM seeds | 89 | 63 | 67 | 70 | 66 |
| 0.75 nM seeds | 110 | 96 | 103 | 119 | 114 |
| **Regulated growth with Ci = 1000 (Supplementary Figure 16)** | | | | | |
| | 8 hr | 24 hr | 32 hr | 48 hr | 72 hr |
| 0.075 nM seeds | 141 | 83 | 88 | 82 | 63 |
| 0.25 nM seeds | 127 | 157 | 109 | 94 | 99 |
| 0.75 nM seeds | 139 | 137 | 110 | 222 | 111 |
| **Regulated growth with Ci = 1250 (Figure 5)** | | | | | |
| | 8 hr | 24 hr | 32 hr | 48 hr | 72 hr |
| 0.075 nM seeds | 131 | 88 | 101 | 100 | NA |
| 0.25 nM seeds | 225 | 106 | 138 | 104 | 118 |
| 0.75 nM seeds | 142 | 142 | 125 | 133 | 147 |
| **Regulated growth with Ci = 1500 (Supplementary Figure 16)** | | | | | |
| | 8 hr | 24 hr | 32 hr | 48 hr | 72 hr |
| 0.075 nM seeds | 181 | 131 | 110 | 125 | 119 |
| 0.25 nM seeds | 113 | 144 | 141 | 108 | 122 |
| 0.75 nM seeds | 103 | 122 | 111 | 148 | 180 |
| **Regulated growth with Ci = 1690 (Supplementary Figure 16)** | | | | | |
| | 8 hr | 24 hr | 32 hr | 48 hr | 72 hr |
| 0.075 nM seeds | 115 | 113 | 127 | 70 | 113 |
| 0.25 nM seeds | 73 | 125 | 97 | 111 | 127 |
| 0.75 nM seeds | 103 | 136 | 129 | 104 | 102 |
| **Figure 6a-c** | | | | | |
| | 6.5 hr | 24 hr | 32 hr | 48 hr | 72 hr |
| S1 regulated | 183 | 93 | 94 | 73 | 64 |
| S1 unregulated 150 nM | 132 | 186 | 53 | 113 | 111 |
| S2 regulated | NA | NA | 107 | 76 | 58 |
| S2 unregulated 150 nM | NA | NA | 1 | 9 | 4 |
| **Figure 6d-f** | | | | | |
| | 7 hr | 23 hr | 32 hr | 48 hr | 72 hr |
| No caps | 116 | 82 | 68 | 58 | 65 |
| 8hr caps | 109 | 112 | 88 | 87 | 38 |
| 24hr caps | 92 | 72 | 78 | 44 | 54 |

*Quantification of concentration of monomers incorporated into nanotubes*

To quantify the concentration of S monomers incorporated into nanotubes in a given sample at a given time point, the total number of nanotube pixels in the qFSwN nanotube image was determined and converted to concentration as follows:

$$(12)\ \ [S\ monomers\ in\ tubes] = \frac{2 * S\ monomers\ per\ pixel * \left(\ hvc\ pixels + \sqrt{2} * dc\ pixels\right)}{volume\ on\ slide * \frac{field\ of\ view\ area}{coverslip\ area} * Avogadro's\ \#} * dilution\ factor$$

Since each pixel that is horizontally or vertically connected to another pixel (*hvc pixels* in Supplementary Equation 12) is 170 nm, each monomer is 14.3 nm[8], and there are 3 S monomers per each row of a nanotube, each *hvc pixel* was considered to have 35.5 S monomers (S *monomers per pixel* in Supplementary Equation 12). Each pixel that is diagonally connected to another pixel (*dc pixels* in Supplementary Equation 12) was considered to have $\sqrt{2}$**S monomers per pixel.* It was assumed that nanotubes bind to the surface of the coverslip and the glass slide with equal probability[3] so the total S monomers in tubes determined from the image of the coverslip was multiplied by 2. Each slide was prepared with 5 µL of the sample (*volume on slide*). The field of view of each image was 87 µm$^2$ and the coverslips we used were 18 mm$^2$ (Methods of the main text). Since the samples were diluted before imaging, the final concentration obtained was multiplied by the dilution factor used for imaging. Plugging all these values into Supplementary Equation 12 yields:

$$[S\ monomers\ in\ tubes] = \frac{(2 * 35.5) * \left(\ hvc\ pixels + \sqrt{2} * dc\ pixels\right)}{5 * 10^{-6}\ L * \left(\frac{0.087\ mm}{18\ mm}\right)^2 * 6.022 * 10^{23} \frac{molecules}{mol}} * dilution\ factor$$

Error bars for the concentration of S monomers incorporated into nanotubes represent the standard deviation of the estimate of the concentration of monomers incorporated made from the single images in a sample.

*Quantification of concentration of seeds*

The concentration of seeds in a given sample can be computed using a previously developed formula[3] similar to Supplementary Equation 12:

$$(13)\ \ [seeds] = \frac{2 * seeds\ per\ field\ of\ view * dilution\ factor}{5 * 10^{-6}\ L * \left(\frac{0.087\ mm}{18\ mm}\right)^2 * 6.022 * 10^{23} \frac{molecules}{mol}}$$

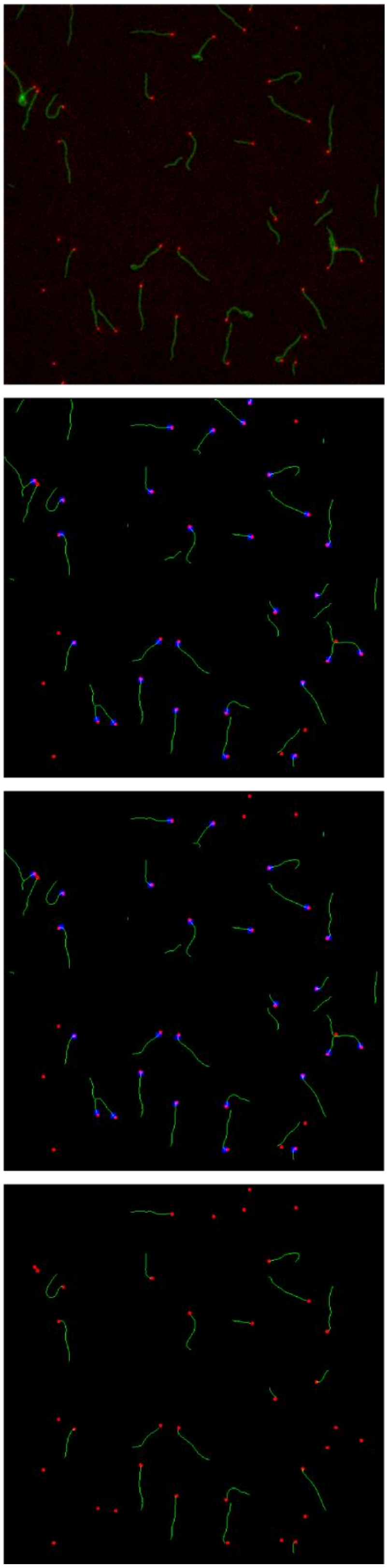

A

Overlaid fluorescence micrographs of nanotubes and seeds to be processed

B

Processed nanotube image to quantify the fraction of seeds with nanotubes (qFSwN) overlaid with its correscponging processed seed image (qS)

C

Processed nanotube image to quantify the fraction of nanotubes with seeds (qFNwS) overlaid with its corresponding processed seed image (qS)

D

Processed nanotube image to quantify nanotube length (qNL) overlaid with its correscponding processed seed image (qS)

**Supplementary Figure 28**: Representative images from the image analysis process used to measure nanotube lengths, fraction of nanotubes with seeds, fraction of seeds with nanotubes and the concentration of monomers incorporated into nanotubes. (**A**) Representative overlaid fluorescence micrograph of the image to be processed. Nanotubes are green and seeds are red. (**B**) Representative overlaid binary image output used to quantify the fraction of seeds with nanotubes. The blue squares indicate the seeds that the algorithm identified as being attached to nanotubes. (**C**) Representative overlaid binary image output used to quantify the fraction of nanotubes with seeds. Nanotubes at the edges of the image in (B) have been removed. The blue squares indicate the nanotube endpoints that the algorithm identified as being attached to seeds. (**D**) The overlaid binary image output analyzed to quantify nanotube length. The branched objects and unseeded nanotubes from the image in (C) have been removed.

**Supplementary References**


1. Mohammed, A. M. & Schulman, R. Directing Self-Assembly of DNA Nanotubes Using Programmable Seeds. *Nano Lett.* **13**, 4006–4013 (2013).
2. Mohammed, A. M., Šulc, P., Zenk, J. & Schulman, R. Self-assembling DNA nanotubes to connect molecular landmarks. *Nat. Nanotechnol.* **12**, 312–316 (2017).
3. Agrawal, D. K. *et al.* Terminating DNA Tile Assembly with Nanostructured Caps. *ACS Nano* **11**, 9770–9779 (2017).
4. Hariadi, R. F., Yurke, B. & Winfree, E. Thermodynamics and kinetics of DNA nanotube polymerization from single-filament measurements. *Chem. Sci.* **6**, 2252–2267 (2015).
5. Gillespie, D. T. Exact stochastic simulation of coupled chemical reactions. *J. Phys. Chem.* **81**, 2340–2361 (1977).
6. Wagenbauer, K. F. *et al.* How We Make DNA Origami. *ChemBioChem* **18**, 1873–1885 (2017).
7. Zhang, D. Y. & Winfree, E. Control of DNA strand displacement kinetics using toehold exchange. *J. Am. Chem. Soc.* **131**, 17303–17314 (2009).
8. Rothemund, P. W. K. *et al.* Design and Characterization of Programmable DNA Nanotubes. *J. Am. Chem. Soc.* **126**, 16344–16352 (2004).
9. Scalise, D., Dutta, N. & Schulman, R. DNA Strand Buffers. *J. Am. Chem. Soc.* **140**, 12069–12076 (2018).
10. Jorgenson, T. D., Mohammed, A. M., Agrawal, D. K. & Schulman, R. Self-Assembly of Hierarchical DNA Nanotube Architectures with Well-Defined Geometries. *ACS Nano* **11**, 1927–1936 (2017).
11. J. Canny. A Computational Approach to Edge Detection. *IEEE Transactions on Pattern Analysis and Machine Intelligence* **PAMI-8**, 679–698 (1986).
